# Experiences, needs and priorities of family caregivers of people with severe mental health conditions in low- and middle-income countries: A systematic review of qualitative studies

**DOI:** 10.1017/gmh.2026.10224

**Published:** 2026-05-13

**Authors:** Dessalegn Enkoyee, Wubalem Fekadu, Awoke Mihretu, Agumasie Semahegn, Siqi Xue, Yodit Tesfaye, Lars Dumke, Anne Marijn De Graaff, Aiysha Malik, Atalay Alem, Abebaw Fekadu, Charlotte Hanlon

**Affiliations:** 1 Department of Psychiatry, WHO Collaborating Centre for Mental Health Research and Capacity Building, Addis Ababa University College of Health Sciences, Ethiopia; 2 Center for Innovative Drug Development and therapeutic Trials for Africa (CDT-Af), Ethiopia; 3 Haramaya University College of Health and Medical Sciences, Ethiopia; 4 Psychiatry, University of Toronto Temerty Faculty of Medicine, Canada; 5 Centre for Addiction and Mental Health, Canada; 6 Mental Health Service Users Association, Ethiopia; 7 Psychiatry and Psychotherapy, University Medical Center Hamburg-Eppendorf, Germany; 8 Department of Noncommunicable Diseases and Mental Health, Mental Health and Substance Use unit, World Health Organization, Switzerland; 9 Global Health and Infection Department, Brighton and Sussex Medical School, UK; 10 Division of Psychiatry, The University of Edinburgh Centre for Clinical Brain Sciences, UK

**Keywords:** families and mental health, caregiver, culture, primary care, schizophrenia

## Abstract

Family caregivers in low- and middle-income countries (LMICs) provide the lion’s share of care for their relatives with severe mental health conditions amid vast treatment gaps. Yet, their lived experiences are not adequately explored. This systematic review synthesizes evidence on the lived experiences, priorities and needs of these caregivers across diverse LMIC settings. We analyzed 76 articles identified across nine databases. Data were synthesized using thematic analysis. The synthesis identified five themes: (1) the journey to understanding, (2) familial commitment to care, (3) the unrelenting burden of caregiving, (4) Forging resilience: strategies of enduring care and (5) voiced needs and priorities. The early attempts to understand the illness take the family on a journey from initial uncertainty to experiential learning. Familial commitment to care is often rooted in moral obligation and system neglect, but this sustained effort leads to an immense caregiving toll. The burden is profoundly gendered, disproportionately affecting women, who commonly face isolation and burnout. Caregivers often navigate pervasive, multidimensional stigma that restricts the entire family’s social and economic future. Despite these challenges, resilience is fostered through faith, peer support and active inclusion of the person in family routines. Caregivers urgently prioritized mental health services that offer knowledge about the illness, active and respectful involvement in treatment planning, practical caregiving skills and support groups. The pressing need for economic support was also expressed. This review underscores the need for global mental health endeavors to recognize and respond to unsupported family caregiving. Family focused interventions have the potential to modify the home environment in ways that support recovery for the person and alleviate many of the caregiving challenges faced by the family. Alongside this, initiatives are needed to address economic precarity and facilitate social inclusion of the family unit.

## Impact statement

This systematic review addresses a critical blind spot in global mental health: the wellbeing of the family caregivers who provide most of the care for people with severe mental health conditions in low- and middle-income countries (LMICs). Drawing on evidence from 76 qualitative studies, it brings caregivers’ voices to the forefront and highlights the immense emotional, social and economic pressures they endure within largely unsupported home environments. The review traces caregivers’ journeys from early confusion and fear to hard-won resilience grounded in faith, peer connections and efforts to maintain the person’s place within family life. It also shows how systemic neglect shifts responsibility for care almost entirely onto families, disproportionately onto women. Importantly, it documents what caregivers say they urgently need: accessible information, involvement in treatment decisions, practical skills, peer support networks and financial relief. By amplifying the voices of caregivers, this review challenges global mental health approaches that rely on family care without investing in structures that sustain it. While existing efforts focus on treating the person with severe mental health conditions, this research demonstrates that the home environment is the primary setting for recovery in many LMICs. The findings make a compelling case for shifting from individual-focused clinical models toward family centered interventions that improve recovery environments at home while reducing caregiver burnout and isolation. They call for approaches that recognize family caregiving as both gendered and economically consequential. Supporting the family unit can be a cost-effective strategy to close the mental health treatment gap in LMICs and advance broader goals of health equity, gender justice and social inclusion.

## Introduction

Severe mental health conditions (SMHCs), such as schizophrenia and bipolar disorder, are often chronic and result in significant impairment in one or more areas of functioning (Vigo et al., 2016; Asher et al., 2017; Forthal et al., 2019; Smartt et al., 2021). Without adequate treatment and support, these impairments are often exacerbated (Fekadu et al., 2019). Contemporary treatment approaches reflect the biopsychosocial understanding of mental illness, incorporating both biological and psychosocial interventions (Gaebel and Zielasek, 2015). However, these interventions are not universally accessible. Globally, the mental health treatment gap is substantial, but it is particularly acute in low- and middle-income countries (LMICs), where up to 90% of individuals may lack effective treatment (WHO Team: Mental Health BH, and Substance Use, 2024).

Research from high-income countries (HICs) has extensively documented the emotional, social and economic burdens faced by caregivers of people with SMHCs and demonstrated the effectiveness of family interventions in reducing burden and improving outcomes (Hayes et al., 2015; Bighelli et al., 2021; Sin et al., 2021; Rodolico et al., 2022). These findings have informed clinical guidelines in HIC settings (NICE, 2014; Ventriglio et al., 2020). However, evidence from HICs cannot illustrate the scale or nature of family caregiving experience in LMICs. In LMIC, contexts with limited social security systems and scarce biomedical mental health services, families provide almost all ongoing care, becoming the de facto primary support (Tirfessa et al. 2019). Cultural norms, family structures, health system constraints and greater reliance on traditional and religious healing practices further shape distinct caregiver experiences (Mendenhall et al., 2014; Brandon and Kohrt, 2015; Patterson et al., 2020).

Although research on caregiver experiences in LMICs is growing, existing reviews have important limitations. Quantitative syntheses offer data on the magnitude of burden but fail to capture nuanced lived experiences and support needs (Andualem et al., 2024). Existing qualitative reviews, meanwhile, are highly restricted in scope. Geographically, these syntheses are largely confined to single regions, such as the Middle East (Alyafei et al., 2021) or sub-Saharan Africa (Ntsayagae et al., 2019). Thematically, other reviews narrowly focused on singular dimensions of caregiving, such as economic burden (Addo et al., 2018; Kisangala et al., 2024), or stigma (Yin et al., 2020), missing the holistic caregiving experience.

Therefore, a critical knowledge gap remains: to our knowledge, no global systematic review has synthesized qualitative evidence on the multifaceted experiences, needs and priorities of caregivers across the diverse cultural and health-system contexts of LMICs worldwide. This qualitative systematic review addresses this gap by synthesizing available qualitative evidence on the lived experiences of family caregivers of people with SMHCs in LMICs. By generating a deeper, cross-contextual understanding, this synthesis aims to provide a foundational evidence base to inform the development of feasible, culturally appropriate and effective family interventions tailored to local LMIC realities.

## Methods

We followed the ENhancing Transparency in REporting the synthesis of Qualitative research statement to enhance transparency in reporting the synthesis of qualitative research (Tong et al., 2012). The protocol was prospectively registered with PROSPERO (CRD420250654264).

### Condition or domain being studied

The domains of interest were: (1) the lived experiences of families caring for a family member with SMHC and (2) families’ perceived needs and priorities for intervention. Family caregivers can be parents, spouses, siblings, adult children or extended family members who provide unpaid, informal care to a relative diagnosed with an SMHC. Studies focusing on nonfamily informal caregivers (such as neighbors, community members or traditional healers) or formal, paid caregivers were excluded. The search terms used to capture the condition of interest, family and the study setting are listed in Supplementary File S1.

We searched the following databases from 2013 up to February 7, 2025: CINAHL, Embase, ERIC (Ebsco), Global Health, Global Index Medicus, PsycINFO, PubMed, Scopus and Web of Science. We included studies published from 2013 onward to align with the adoption of the first WHO Comprehensive Mental Health Action Plan (2013–2030) (WHO Team: Mental Health BH, and Substance Use (MSD), 2021). This timeframe allowed us to capture recent evidence that was not included in previous reviews. The search strategy combined controlled vocabularies and keywords related to severe MHCs (e.g., “severe mental illness,” schizophrenia); family caregivers (e.g., parent, spouse, caregiver), qualitative research (e.g., qualitative study, opinions, meanings) and standard terminology for LMICs, as defined by the World Bank (2024). There were no search restrictions by language. The full search strings used are provided in Supplementary File S1.

### Eligibility criteria

We included primary qualitative studies (i.e. ethnography, phenomenology, grounded theory) that utilized established qualitative methods for data collection and analysis (such as thematic, phenomenological or content analysis). Participants included family caregivers of individuals diagnosed with SMHCs, regardless of the duration of the illness. For this review, SMHCs were operationally defined to include affective and nonaffective psychoses (schizophrenia and bipolar disorder), as well as broad classifications of ‘severe mental illness’ as defined by the authors of the primary studies. Eligible studies focused on caregiving experiences, coping mechanisms, challenges and support needs within LMICs. We excluded book chapters, reviews, editorials and conference abstracts.

### Data extraction and study quality assessment

All retrieved citations were imported into Rayyan software (Ouzzani et al., 2016), where duplicates were removed. Title and abstract screening was conducted as a single-rating process, with the total yield of citations divided among five independent reviewers. To ensure high consistency and mitigate the limitations of single screening, the team held extensive calibration meetings before screening to align on the eligibility criteria. Reviewers adopted a conservative, inclusive approach during this phase; any citation deemed ambiguous or unclear was automatically retained for full-text assessment. Full-text articles of potentially relevant studies were subsequently retrieved and assessed for final eligibility. Uncertainties or disagreements were resolved through team discussion and consultation with a senior reviewer. Reasons for exclusion at the full-text stage were documented in Rayyan and reported in the PRISMA flow diagram.

To further ensure methodological rigor and verify that the eligibility criteria were applied consistently across the team, we conducted a collaborative pilot of the data extraction process. All five reviewers independently extracted data from the first 20% of the included studies using a collaboratively developed, standardized extraction sheet. The team then held a consensus meeting where reviewers presented their extractions, discussed their experiences and resolved any challenges. This exercise not only refined the usability of the extraction tool but also served as a rigorous check of our shared understanding of the inclusion criteria. After finalizing the extraction sheet, individual reviewers extracted data from their assigned remaining studies. The prepiloted form captured key study characteristics, including author, year of publication, country, aim, population, phenomenon of interest, context, methods and main findings.

The methodological quality of the included studies was independently assessed by the reviewers using the Joanna Briggs Institute (JBI) Critical Appraisal Checklist for Qualitative Research (Lockwood et al., 2015). This assessment evaluated the alignment between research methods and questions, the representation of participants’ voices and the rigor of data analysis. While this appraisal informed our overall interpretation of the findings, no studies were excluded based on quality scores alone. The detailed rating for each item on the quality appraisal checklist is presented in Supplementary File S2.

### Strategy for data synthesis

All included full-text articles were uploaded to NVivo (version 14) for data management and analysis. To clarify the scope of our data extraction, we specifically coded the “Findings/results” sections of the included articles. This encompassed both first-order constructs (direct quotations from family caregivers) and second-order constructs (the primary authors’ analytical interpretations and summaries).

We conducted a thematic synthesis following the established approach described by Thomas and Harden (2008). Our interpretation was guided by a phenomenological

theoretical framework to ensure the synthesis remained grounded in the nuanced, lived experiences of the participants. The synthesis involved three distinct stages:

1. Line-by-line coding: The primary reviewer applied inductive codes to the extracted text.
2. Development of descriptive themes: These initial codes were grouped into broader, descriptive categories based on similarities and shared meanings.
3. Generation of analytical themes: We synthesized the descriptive categories to generate new interpretative findings that directly addressed our research question.

To ensure analytical rigor and depth, two reviewers shared the coding responsibilities. Initially, these two reviewers independently coded a subset of 10 articles and subsequently held a consensus meeting to compare their codes and processes. This collaborative exercise was designed to enrich the coding process. By combining their complementary perspectives, the reviewers were able to capture a more comprehensive and nuanced range of codes during the initial phase.

This exercise informed the development of a robust initial codebook. The two reviewers then utilized this codebook to code the remaining articles, holding frequent discussions with the wider research team to refine codes and resolve any discrepancies. Finally, a lived-experience expert, who is a member of the review team, reviewed the categories and analytical themes, contributing their insights to the final interpretation of the findings.

### Authors’ reflexivity

Throughout the review process, the research team engaged in continuous reflexive practice to acknowledge and mitigate how our respective biases and positionalities influenced data interpretation. While our funder provided feedback on methodological milestones through weekly and biweekly meetings, they had no role in data analysis, theme generation or the interpretation of findings, ensuring the team’s analytical independence.

Our core team comprised individuals with diverse backgrounds in psychology, psychiatry, mental health systems research and lived experience of psychosis. We explicitly recognized that our clinical backgrounds (psychology and psychiatry) inherently predisposed us to view caregiver burden through a biomedical lens, potentially leading us to overemphasize symptom management and clinical deficits. To counter this, team consensus meetings were used to actively challenge these clinical biases. These deliberations helped us ensure that the sociocultural, economic and structural realities of caregiving in LMICs were elevated in the coding process. The mental health systems researchers on our team helped contextualize these burdens within the broader landscape of LMIC health system constraints.

## Findings

### Characteristics of included studies

**Figure 1.**
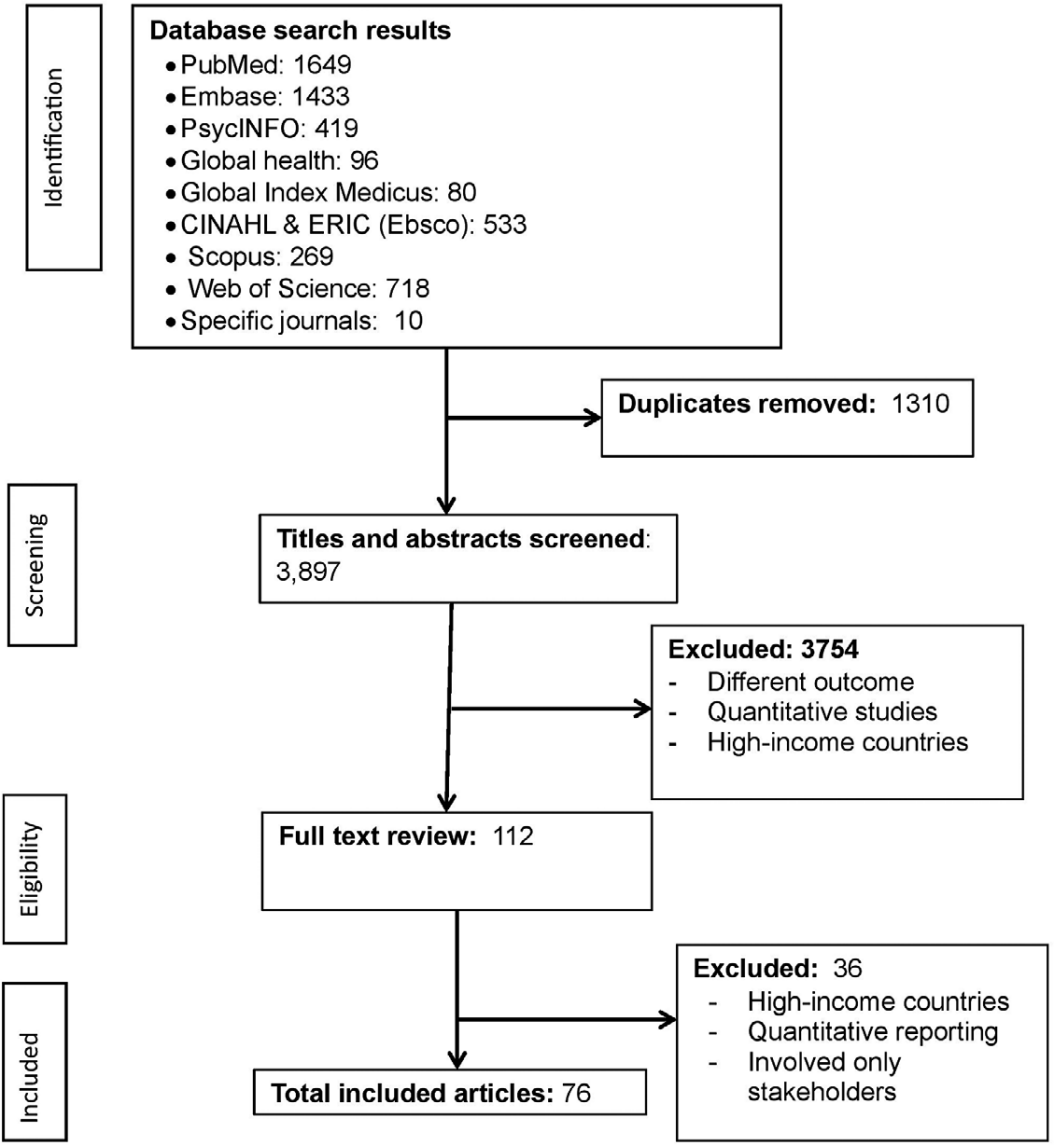
PRISMA flow diagram. Figure 1. long description.

A total of 76 (*n* = 76) articles met the inclusion criteria (Figure 1). Descriptions of all included studies are presented in Supplementary File S2. The included studies covered a range of geographical and cultural settings, originating from all six WHO regions (Table 1). The largest number of studies came from the South-East Asia Region (20 studies), followed by the Eastern Mediterranean Region (17 studies), the African Region (14 studies), the Western Pacific Region (11 studies), the European Region (8 studies) and the Region of the Americas (6 studies).

**Table 1.** Number of articles included in the review from the WHO regions Table 1. long description.

WHO region	Countries included	Total studies
South-East Asia Region (SEAR)	India, Indonesia	20
Eastern Mediterranean Region (EMR)	Iran, Oman, Pakistan	17
African Region (AFR)	Ethiopia, Ghana, Nigeria, South Africa, Tanzania, Uganda, Zimbabwe	14
Western Pacific Region (WPR)	China, Malaysia, Thailand	11
European Region (EUR)	Turkey	8
Region of the Americas (AMR)	Brazil, Colombia, Mexico	6

Among the 76 included studies, eight focused on a single-family caregiver group such as mothers (*n* = 2), spouses (*n* = 1), siblings (*n* = 3) or children (*n* = 2). The rest of the studies included a mix of two or more family member types, such as a mix of parents, spouses and siblings, some extending to children or other relatives. The majority of the included articles (72.2%, *n* = 55) focused on participants diagnosed with schizophrenia. Smaller proportions of the studies included individuals with bipolar disorder (13.9%, *n* = 10) or participants broadly categorized as having a severe mental illness (13.9%, *n* = 11). The included articles were conducted across a variety of settings. One-third of the articles (33.3%) reported data collected in outpatient psychiatric services, 30.2% in community settings, 22.2% were in inpatient psychiatric facilities and 14.3% of studies on mixed settings that combined both community and clinical environments (Supplementary File S2).

Sixty-one articles (80%) used in-depth interviews as the primary data collection method, while seven studies used focus group discussions. A small number of studies employed combined methods (*n* = 7), and only one study utilized observation as a data collection approach. Across the included studies, thematic analysis was the most frequently used analytical approach (30 studies), followed by content analysis (18 studies). A smaller number of studies applied Interpretative Phenomenological Analysis (7 studies), Framework analysis (4 studies) and Grounded Theory (4 studies). The remaining studies (*n* = 13) used other or unspecified qualitative analysis methods.

Quality appraisal using the JBI Critical Appraisal Checklist for Qualitative Research indicated that most included studies (85%, *n* = 65) were rated “YES” on items assessing coherence between the stated philosophical perspective, research methodology, research questions, data collection, analysis and interpretation of findings (Supplementary File S2). In contrast, aspects of researcher reflexivity were consistently underreported. Only 30% (*n* = 23) of studies explicitly described the researcher’s cultural or theoretical positioning, and fewer than one-third (25%, *n* = 19) addressed the potential influence of the researcher on the research process and participants (Supplementary File S2).

### Overview of themes

**Figure 2.**
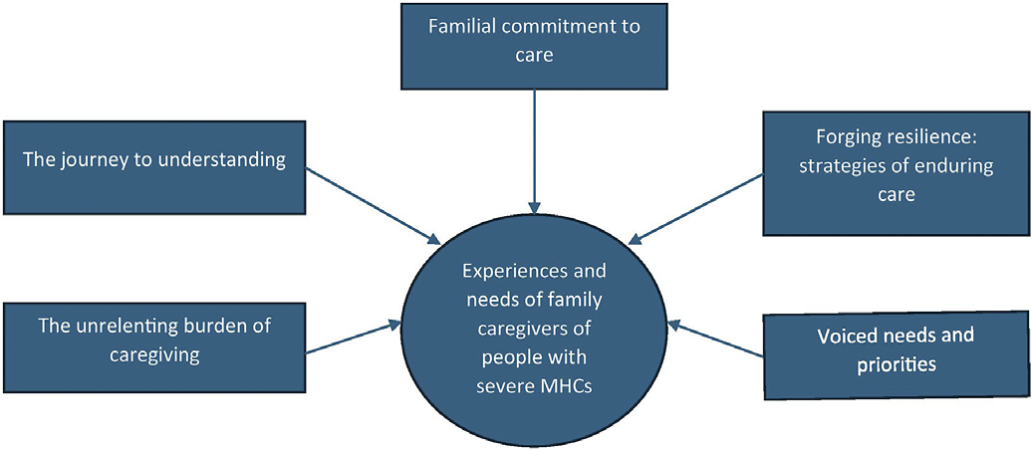
Conceptual map of the themes describing the experience of family caregivers of people with severe mental health conditions in LMICs.

Family caregivers described a multifaceted caregiving journey shaped by their evolving understanding of the illness, daily caregiving demands and the social and structural environments in which care took place. Thematic synthesis yielded five interconnected themes (Figure 2): (1) the journey to understanding the illness, (2) family commitment to care, (3) the unrelenting burden of caregiving, (4) strategies to forge resilience and (5) voiced needs and priorities. Initially, family caregivers embarked on a complex journey to make sense of the condition, which subsequently shaped their deep commitment to providing care. However, assuming the lion’s share of this responsibility exacted a significant emotional, social and economic toll on these families. Despite these profound challenges, family caregivers developed coping mechanisms and resilience to sustain both themselves and their relatives with SMHCs. Finally, emerging from these complex lived experiences are the clearly defined support needs and priorities of the family caregivers. The following sections provide a detailed narrative account of each theme.

#### Journey to understanding the illness

This theme describes the dynamic and laborious process families undergo to make sense of the illness, moving from deep confusion to hard-won, experiential expertise. The caregiving journey typically begins with uncertainty, as families struggle to name and interpret bewildering symptoms, often turning to spiritual or traditional explanations (Olwit et al., 2015; Khadem et al., 2022; Dijkxhoorn et al., 2023). As one mother recalled, *“*People said he was bewitched… I was in a state of confusion” (Olwit et al., 2015). Early signs are commonly misinterpreted as personal failings, and this disorientation is intensified by the systemic failure of the formal (biomedical) health system to provide clear information or nonjargon explanations, leaving families isolated and unprepared (Bellini et al., 2016; Pan et al., 2024). As one caregiver lamented, “No, he [the doctor] never clarified it to me… Neither there, nor here, nor anyone [explained]” (Bellini et al., 2016).

Faced with this informational void, families embarked on a desperate search for help. They navigated a fragmented landscape that spans biomedical facilities, traditional healers and religious institutions (Nuraini et al., 2021; Verity et al., 2021). The hospital often serves as a last resort. This search itself is costly and tiring, forcing caregivers into a role they are unprepared for: “I had to take on the role of family caregiver without being ready” (Pan et al., 2024). Over time, this self-directed effort fosters a pragmatic, experiential expertise in daily management, characterized by active problem-solving. Families start to adjust routines, define roles and responsibilities and involve the person with the illness (Akgül Gök and Duyan, 2020; Heydarikhayat et al., 2025): *“*Once he takes his medicine … his brothers take him to his job” (Heydarikhayat et al., 2025).

Receiving a diagnosis often brings devastating news, unleashing grief, anxiety and despair that can even push caregivers to contemplate self-harm (Bai et al., 2020; Heydarikhayat et al., 2025). This sorrow is primarily centered on mourning the loss of the future envisioned for their family member, such as missed educational milestones, career prospects and opportunities to build their own family (Liu and Zhang, 2020; Fekadu et al., 2024; Pan et al., 2024). A father mourned, *“*She was a very good student… I would have grandchildren by now” *(*Fekadu et al., 2024 *).* For primary caregivers who are spouses of the person, the change in roles fundamentally transforms relationships, often replacing romantic love with a sense of compassionate duty or pity: (Rahmani et al., 2018; Hasanpour et al., 2024) “My love… turned to this feeling of pity… for the sake of God” (Rahmani et al., 2018).

#### Familial commitment to care

This theme focuses on the profound motivation that sustains caregiving. At the core of caregiving lies a strong moral imperative, kinship and communal duty to sustain efforts amid sacrifices and absent reciprocity. Caregiving is not framed as a choice but as an inherent, defining moral duty attached to the caregiver’s identity as a parent, sibling or spouse (Liu et al., 2022). It transcends personal wellness: “How could I give up? He is my son. I have a duty” (Liu et al., 2022). External social pressure reinforces this perceived duty, as failing to provide care can lead to harsh community judgment and social condemnation (Read and Nyame, 2019). One caregiver explained: “……People will be talking against you… you are not trying your best” (Read and Nyame, 2019).

Commitment to care often manifests in exhausting labor; endless vigilance over meals, hygiene, medications and ensuring protection from harm. One participant described the scope of their duties: “I have to do everything for my son… supervise everything from eating to taking medicine” (Tamizi et al., 2020). The continuous supervision severely limits caregivers’ mobility, autonomy and capacity to earn an income, resulting in lost livelihoods and consumption of personal time (Gloria et al., 2018; Demissie et al., 2021). The cost of caregiving often has a ripple effect, leading to neglect of other healthy family members (Karaca et al., 2024). One study noted the children of caregivers often “received little care or interest from the healthy parent who undertakes the care of their spouse and therefore is tired and fails to care for their children” (Karaca et al., 2024).

To endure these pressures, families actively negotiate responsibility. Support gathered manifests as role sharing, shared emotional burden and long-distance practical aid from extended relatives (Amaresha et al., 2019; Heydarikhayat et al., 2025; Sari and Duman, 2025). A caregiver shared, “Whenever I fall into despair, my elder sister… gets me into shape” (Sari and Duman, 2025). However, this negotiation is highly gendered, with the primary, day-to-day emotional and practical work falling almost exclusively to women, particularly mothers (Attepe Özden and Tuncay, 2018). One mother stated, “All in all, the father works. He goes in the morning and returns in the evening. In addition, our child is female. It’s me [who] cares more” (Attepe Özden and Tuncay, 2018). This structural dynamic often results in mothers bearing the brunt of day-to-day care, transforming the “family” unit into the “mother” alone (Read and Nyame, 2019; Kanungpiarn et al., 2021). Lack of support can also be fraught with conflict, where pleas for help are met with criticism rather than assistance: “My husband… blames me, saying I’m not doing a good enough job” (Pan et al., 2024). Another mother felt her only remaining support was divine: “I have no man except God” (Read and Nyame, 2019).

#### The unrelenting burden of caregiving

The caregiving role can exact a substantial and multidimensional toll that threatens the caregiver’s emotional stability, financial security and social life. For many, the emotional landscape is characterized by a relentless cycle of worry, sadness, frustration and guilt (Jack-Ide et al., 2013; Marimbe et al., 2016). A caregiver shared their experience, “I have sleepless nights… worsened my high blood pressure” (Jack-Ide et al., 2013). The daily grind of repetitive, often unappreciated tasks fuelled a sense of helplessness and led to a feeling of being trapped in the caregiving cycle (Bedoya Hernandez and Builes Correa, 2013; Wulandari et al., 2024). Erosion of identity and a paradoxical mix of deep love and resentment were reported as a result of the constant pressure of caregiving. Families expressed anger and guilt that are directed both inward and outward (Saville Young and Flannigan, 2021; Kalayci et al., 2023). Chronic strain shatters the peace of the home, leading some to regret their relational choices or contemplate divorce (Asher et al., 2017; Kargar et al., 2021). For some, the exhaustion and hopelessness reach a point where they express desperate wishes for the death of their loved one, or contemplate ending their own lives, compounded by deep anxiety over the future of their family member with psychosis (Peng et al., 2022; Lohrasbi et al. 2024; Soni and Kumar, 2024).

Caregivers described a sense of alienation from their relative. A spouse reported feeling as though she was “dealing with a stranger” (Al-Sawafi et al., 2021). Intimacy was transformed into functional exchanges: “I can’t communicate…[I] just give him the medicine” (Rahmani et al., 2018). In some families, communication breakdowns fuelled irritability that further strained family interactions. One participant acknowledged this cycle: “I get easily irritated …‥and that affects my interactions with people” (Mbadugha et al., 2023). Paradoxically, crises are reported to also deepen bonds: “After I became sick, I… bared my heart to my mother… Now, we had a good relationship” (Attepe Özden and Tuncay, 2018).

The caregiving burden indicates not only personal strain but also the absence of support, often driving families into financial collapse. The simultaneous difficulties faced by the person in work and the caregiver’s need to provide constant supervision can force the caregiver to abandon their livelihoods, plunging families into debt and precarity: “Now I don’t work. All my time is used up” (Soni and Kumar, 2024). Economic devastation accompanied a loss of social connection. For some families, the all-consuming nature of caregiving led to total confinement and isolation, trapping caregivers and forcing younger family members to abandon their own aspirations to support the household (Tamizi et al., 2020; Saville Young and Flannigan, 2021; Heydarikhayat et al., 2025). One sibling lamented, “when my mom got sick, I became their mother” (Heydarikhayat et al., 2025). For some, the erosion of oneself felt like being “a butterfly who has been trapped in a spider’s web” (Rahmani et al., 2018).

The unpredictability of the illness in some individuals necessitated a state of constant hypervigilance, transforming the home into a site of potential danger (Gloria et al., 2018; Kanungpiarn et al., 2021; Hasanpour et al., 2024). Caregivers reported living with the constant, often realized, threat of physical violence, verbal abuse and property destruction (Demissie et al., 2021; Neha et al., 2021; Nuraini et al., 2021; Putri et al., 2022). Managing high-risk behaviors defined the caregiving experience for many. In the absence of external support, families often resort to desperate and coercive measures like physical restraint to prevent harm (Verity et al., 2021; Ilmy et al., 2022).

Stigma is another pervasive burden that imposes social isolation and exclusion. Caregivers may internalize society’s negative attitudes, leading to deep feelings of shame and embarrassment (Read and Nyame, 2019; Akgül Gök and Duyan, 2020; Sawab et al., 2023). Families protect themselves from ridicule and social condemnation by employing a conscious strategy of concealment (Al-Sawafi et al., 2021; Heydarikhayat et al., 2025). Disclosure often leads to social rejection and isolation, where family and friends withdraw support (Chen et al., 2019; Karaca et al., 2024). Most consequentially, stigma creates a deep fear for the future, tainting the reputation of the entire family and actively hindering the marital and professional prospects of healthy siblings (Kargar et al., 2021; Latifian et al., 2022; Heydarikhayat et al., 2025). While families often stood as a united front against public stigma, the home was not always a safe space for people with severe MHCs. Exclusion within the home is often manifested as a lack of genuine intimacy and connection with the person (Rahmani et al., 2018), active distancing by relatives who are not primary caregivers (Kalayci et al., 2023) and avoidance of the person in social events (Latifian et al., 2022).

#### Forging resilience: strategies of enduring care

Family caregivers actively forged diverse coping pathways characterized by psychological adjustment, resource mobilization within the family and tenacious commitment. A foundational strategy involved a cognitive shift toward pragmatic acceptance of the enduring nature of the illness, which is framed as a necessary step to reduce anger and denial (Hailegabriel and Berhanu, 2023; Pan et al., 2024). Active problem-solving and the search for knowledge were born out of this acceptance (Akgül Gök and Duyan, 2020). For many, acceptance is deeply intertwined with religious faith, which provides a framework for understanding suffering, offers emotional solace through prayer and allows caregivers to view their role as a divine test (Iseselo et al., 2016; Gumilang, 2023). The process often involves lowering expectations for a complete cure, providing a measure of peace (Kanungpiarn et al., 2021; Pan et al., 2024). For example, in Thailand, this was framed as *tamjai* (reconciliation to a situation), a conscious adjustment of one’s mindset to the reality of lifelong caregiving, guided by the philosophy that “Whatever happens, happens” (Kanungpiarn et al., 2021).

Resilience is actively fortified through relational resources, especially connecting with peers who share the lived experience (Bademli and Lök, 2020; Liu and Zhang, 2020). As one young caregiver explained: “…There is no one who understands me, there are attendants [family caregivers] in the hospital, it makes me feel good to talk to them. Because we understand each other …” (Bademli and Lök, 2020). Furthermore, families actively seek to involve the person in daily routines and adapted work. This strategy served both as a therapeutic endeavor to counter idleness and relapse and as a powerful means of cultivating a renewed sense of purpose (Budiarto and Mustikasari, 2024; Fitryasari et al., 2024). Involving the person in tailored tasks is reported to affirm the individual’s value as a contributor to the family (Yunita et al., 2020; Fitryasari et al., 2024; Heydarikhayat et al., 2025; Sari and Duman, 2025).

Despite the challenges, the shared struggle is reported as a catalyst for personal growth, deepened relational bonds and empathetic transformation (Bellini et al., 2016; Saville Young and Flannigan, 2021; Mbadugha et al., 2023). A sibling shared his experience: “… I would say his illness brought me close to him.” (Saville Young and Flannigan, 2021) Caregivers reported gaining a deeper insight into mental illness and developing an appreciation for small, incremental progress, finding gratitude and companionship where others might only see burden (Wulandari et al., 2024). A mother said: “….She[her daughter] didn’t want to take a shower or get out of bed, but all praise to God, now….she can wipe her own pants” (Wulandari et al., 2024).

While families demonstrate remarkable resilience, some coping mechanisms born of desperation can become maladaptive. Two prominent maladaptive pathways that emerged from the data were the inequitable and unsustainable burden of gendered care and the use of coercive, overcontrolling measures to manage risk. Leaving the brunt of care to women risks burnout of the primary caregiver and can create fractured, blaming family dynamics (Read and Nyame, 2019; Pan et al., 2024). The desperate use of coercive and overcontrolling measures such as constant scrutiny, forced medical compliance or physical restraint was reported as counterproductive. It often resulted in renewed conflict, shattered trust and inflicted further trauma (Soni and Kumar, 2024; Heydarikhayat et al., 2025).

#### Voiced needs and priorities

Family caregivers expressed a strong need for broad and consistent support in several key areas. They prioritized getting clear and practical information about the illness: what it is, what causes it, how it might progress over time and how it can be treated (Amaresha et al., 2015; Kumar et al., 2019). A caregiver shared… “Don’t know how long he has to take tablets” (Amaresha et al., 2015). Caregivers need specific training on practical management skills, including effective communication strategies to de-escalate difficult situations and tips for recognizing early warning signs (Amaresha et al., 2015; Özgönül and Bademli, 2022). A brother asked, “I know he has a problem, but I cannot control myself… I need some tips in talking with my brother patiently” (Amaresha et al., 2015). Their informational needs also include guidance to counter misinformation within the family and address concerns such as whether the illness may be hereditary (Amaresha et al., 2015).

Beyond simply receiving information, families called for their active and respectful participation in the care and treatment process (Asgari et al., 2023). They expressed their frustration with a healthcare system that sidelines their expertise and excludes them from critical decision-making (Akgül Gök and Duyan, 2020). They demanded a collaborative relationship where their unique insights are valued, and they are not instrumentalized or deceived by professionals (Zarei et al., 2021). A participant described being directed by a physician to deceive their mother: “Do not use a child as a tool to take a mother to a physician. The physician should not use the child and ask him/her to tell lies” (Zarei et al., 2021).

Family caregivers also articulated the need for psychosocial support to combat the deep sense of isolation stemming from community and extended family withdrawal (Fauziah et al., 2024). One caregiver shared their plea for solidarity from their kin: “Well, we want our relatives to be able to provide support, don’t just ignore it, besides being ignorant, avoid it too” (Fauziah et al., 2024). The absence of solidarity in some families not only increased the workload of primary carers but also added emotional strain. A powerful, recurring need was for peer support groups, which were seen as essential for mutual understanding, reducing alienation and building solidarity (Vargas-Huicochea et al., 2018). Systemic needs include calls for professional counseling, legal support and broader interventions to fight societal stigma (Oz et al., 2022; Hailegabriel and Berhanu, 2023).

Another need expressed was robust financial assistance to mitigate the crippling economic burden (Amaresha et al., 2015; Zarei et al., 2021; Hailegabriel and Berhanu, 2023). The financial strain arises from the simultaneous loss of income and the high cost of effective treatment, often forcing families to choose between basic necessities and medication (Amaresha et al., 2015; Hailegabriel and Berhanu, 2023). Caregivers called for financial solutions from governmental or nongovernmental bodies, advocating specifically for support that enables access to affordable and better medications with fewer side effects (Chen et al., 2019; Lohrasbi et al. 2024).

## Discussion

This systematic review synthesized evidence from 76 qualitative studies, offering an understanding of the family caregiving experience for people with severe MHCs in diverse LMIC contexts. Our review consolidates evidence that outlines a complex caregiving journey, most often characterized by moral commitment, with substantial personal burden, systemic resource scarcity, and, for some, emerging resilience. We also highlight the differential impact of caregiving on family members and pinpoint key needs to guide global mental health efforts.

Our findings illustrated a wide range of experiences associated with the caregiving role. Caregivers’ efforts to understand the illness, which involved moving from initial uncertainty to learning through lived experience, align with existing literature (Kuipers et al., 2002). In many of the studies included in this review, families shouldered most of the care responsibilities in the absence of adequate formal support. This heavy reliance on family care often led to competing demands between caregiving and livelihood, contributing to financial strain, job loss and emotional exhaustion. The cumulative social and economic pressures experienced by families in these settings were often more severe than those described in high-income contexts (Attepe Özden and Tuncay, 2018; Pedersen et al., 2019; Abayon et al., 2024; Oluwole and Obadeji, 2025). Across many LMIC settings, strong family ties and a deep sense of moral and relational duty were central in sustaining care for people with severe MHCs. However, the nature and intensity of this commitment can vary across cultural, social and economic contexts (Brandon and Kohrt, 2015). The findings showed how the context of caregiving transforms an otherwise universal family value into a potentially overwhelming obligation.

Our review also highlighted the heavily gendered nature of caregiving. Mothers and other female relatives were routinely expected to shoulder most responsibilities, while men were typically portrayed as less involved in daily care tasks. A recent review from Ethiopia also reported similar findings (Tesfaye and Demelash, 2025). This imbalance can lead to caregiver burnout and, in some cases, strain family relationships or stability (Sharma et al., 2016; Attepe Özden and Tuncay, 2018). However, there are reports where caregiver gender is not always a significant predictor of negative caregiving outcomes (Sharma et al., 2016). Nevertheless, the accounts of family caregivers in this qualitative review are overwhelmingly from female caregivers. Social expectations, cultural norms and limited formal support could place disproportionate responsibility on women. Interventions that fail to account for these gendered dynamics risk overlooking a key source of fragility in the family based care in many LMIC settings. Future initiatives should therefore aim to better support women caregivers and promote more equitable sharing of caregiving responsibilities.

Across the studies, stigma emerged as a deeply embedded and multidimensional force, yet its mechanisms showed specific variations compared to HIC literature. While stigma by association is well-documented in HICs (Corrigan and Miller, 2004), our review highlighted how stigma in LMIC settings often threatens the social capital of the entire family unit. Its effect ranges from internalized shame, community rejection, to institutional barriers that constrained education, employment and marriage opportunities for the entire family (Schomerus and Angermeyer, 2008). In many contexts, families described living under the weight of social judgment, where fear of being identified with a mental illness fostered secrecy and delayed help-seeking. Although the expression and consequences of stigma can vary across cultural and economic settings, its effects are consistently detrimental. Addressing stigma, therefore, requires locally grounded strategies that engage with community leaders and recognize the intersection of social exclusion and economic poverty.

Despite the challenges in caregiving, families often demonstrated remarkable resilience. Caregivers drew on faith, peer connections and structured daily routines to find meaning, sustain hope and strengthen family bonds. Such adaptive strategies countered the dominant language of “burden” with stories of growth and purpose within adversity (Saville Young and Flannigan, 2021). However, this resilience coexists with distressing reports of coercion, restraint and concealment. These responses were often signals of desperation in the face of overwhelming circumstances and limited alternatives. Coercive measures underscore the urgent need for accessible, humane and adequately resourced care options that enable families to protect both their relatives’ dignity and their own wellbeing (Onwumere et al., 2018).

The lived experiences described above reinforce the need for supportive, family focused interventions. Our synthesis identified several intervention targets grounded in caregivers’ realities. A primary gap is basic, comprehensible information about the illness: its nature, causes, likely course and treatment options. Participants in the included studies expressed a need for collaborative, respectful care with jargon-free education about the illness. This needs to focus on practical skills, like symptom management, relapse prevention and conflict resolution (Sin et al., 2017). Health care providers must also address maladaptive coping, such as physical restraint or concealment. Alongside access to mental health care, families could be equipped with alternatives to coercion, such as de-escalation techniques (Onwumere et al., 2018). Families emphasized the importance of being treated as partners whose lived knowledge is valued. Involving them meaningfully in treatment planning and discharge decisions has been shown to improve medication adherence and reduce relapse rates (Chatterjee et al., 2014).

Caregivers also highlighted the value of strengthening relational resilience and the benefits of family to-family networks and support groups. These can alleviate isolation, promote shared learning and provide emotional uplift (Bademli and Duman, 2014). Equally critical are access to available opportunities with no barriers and providing financial support. The economic devastation from caregiving demands more than education. Policymakers need to recognize that reliance on family for all caregiving drives poverty. Solutions like disability grants could cover income gaps and medication costs (Parker-Grewe, 2017). A recent comprehensive review commissioned by UNICEF reported that cash transfer programs for families in Africa increased hopefulness, decreased feelings of shame and greater autonomy (Novignon et al., 2025). Integrating support with income-generating programs or vocational training, especially for women, might reduce dependency on the care recipient’s finances. Finally, legal protections are essential. These should safeguard the assets and rights of both the person with psychosis and their caregivers, particularly in marital or property disputes.

### Strengths and limitations

Strength of this review is the inclusion of 76 studies exclusively from LMICs, providing the necessary depth and contextual focus to inform global policy. The use of thematic synthesis allowed us to move beyond descriptive summaries to generate high-level, analytical themes of commitment, burden and resilience. Furthermore, the inclusion of a multidisciplinary research team ensured that our interpretations remained deeply grounded in the realities of caregiving rather than relying solely on clinical or systemic lenses.

However, several limitations must be acknowledged. First, grouping vastly diverse nations under the broad economic designation of “LMICs” inherently masks significant intra- and inter-regional differences in culture, religion, family structures and health system infrastructures. While our synthesis identified common, cross-cutting themes, local adaptation and cultural tailoring remain essential before applying these findings to specific intervention designs. Second, as noted in our quality appraisal, many of the primary studies lacked explicit researcher reflexivity. The failure of primary authors to interrogate their own theoretical or cultural positioning may have influenced the depth and framing of the original data we extracted. Third, the dominant qualitative narrative in the primary literature is heavily deficit-focused. Because research questions frequently center on “burden” and “challenges,” the positive dimensions of caregiving are likely underrepresented in this synthesis. Finally, the literature search for this review concluded in February 2025. While this represents a comprehensive synthesis of the qualitative literature up to that point, studies published in the subsequent months are not reflected in this analysis; however, given the large volume of included studies (*n* = 76), thematic saturation was robustly achieved.

## Conclusion

Evidence from diverse LMIC settings finds common themes of caregiving as a tenacious moral commitment sustained despite systemic neglect and pervasive stigma. While social and economic assistance remains important, family caregivers also emphasized that access to quality mental healthcare would make their situations manageable. In particular, family focused interventions and consistent clinical guidance were seen as essential yet often unavailable. Without these supports, the financial pressures, social isolation and caregiver exhaustion can become even more severe. A more effective response requires strengthening routine mental health services, providing targeted support to families and addressing the wider social and economic conditions that shape the caregiving experience.

## Supporting information

10.1017/gmh.2026.10224.sm001 Enkoyee et al. supplementary material Enkoyee et al. supplementary material

## Data Availability

The data that support the findings of this study are available from the corresponding author, DK, upon reasonable request.

## Supplementary material

The supplementary material for this article can be found at http://doi.org/10.1017/gmh.2026.10224.

## Figure 1. Long description

At the top, a box lists database search results: PubMed 1649, Embase 1433, PsycINFO 419, Global health 96, Global Index Medicus 80, C I N A H L and E R I C (Ebsco) 533, Scopus 269, Web of Science 718, Specific journals 10. An arrow points right to a box: Duplicates removed 1310. A downward arrow leads to Titles and abstracts screened 3897. To the right, a box: Excluded 3754, with reasons: Different outcome, Quantitative studies, High-income countries. Downward arrow to Full text review 112. To the right, a box: Excluded 36, with reasons: High-income countries, Quantitative reporting, Involved only stakeholders. Downward arrow to Total included articles 76. The left margin labels each stage: Identification, Screening, Eligibility, Included.

Navigate back to Figure 1..

## Table 1. Long description

From the top row, South-East Asia Region includes India and Indonesia with 20 studies. Next, Eastern Mediterranean Region covers Iran, Oman, and Pakistan with 17 studies. African Region lists Ethiopia, Ghana, Nigeria, South Africa, Tanzania, Uganda, and Zimbabwe with 14 studies. Western Pacific Region includes China, Malaysia, and Thailand with 11 studies. European Region contains Turkey with 8 studies. Region of the Americas includes Brazil, Colombia, and Mexico with 6 studies. The table shows a descending trend in study count from South-East Asia Region to Region of the Americas.

Navigate back to Table 1..
