# Supplementary material for: Experiences, needs and priorities of family caregivers of people with severe mental health conditions in low- and middle-income countries: A systematic review of qualitative studies
Source: Glob Ment Health (Camb). 2026 May 13;13:e115. doi: 10.1017/gmh.2026.10224 (PMC13244235; doi:10.1017/gmh.2026.10224)
Supplement: Enkoyee et al. supplementary material [file S2054425126102246sup001.zip › supplementary file 3_themes and exemplar quotes.docx]

Table. Themes and exemplar quotes

| Themes | *Exemplar quotes* |
| --- | --- |
| **The journey to understanding the illness** | *I felt so bad, so sad. I am a single mother and this child has been helping me. I was so devastated. People said he was bewitched and others said probably it was because of cannabis, I was in a state of confusion. (Mother, caretaker, in-depth interview)(Olwit et al. 2015)*  *I don’t think it is common for people to go to the hospital when mental illness happens. Even me personally, on the first onset of mental illness in my patient, I did not think of hospital; we first locked him up for some time and did everything there. Then we invited the Imam (Muslim cleric) to pray for him and he improved for almost a year. But when he broke down again, we decided to come to the hospital.(FGD, caregivers, Kampala)(Verity et al. 2021)*  *Over time, participants cannot deny the existing problems in the patients anymore, however, they are still unable to comprehend what the patients with BD experience. Hence, they fail to fully understand them and their own interaction with them, and instead, they experience a series of changes often inexplicable to them. "I cannot explain it at all, not even to myself. I feel he is not there; I really don’t know what to call it. He is not like what he should be" (C.2).(Khadem et al. 2022)*  *The doctor called me, we talked ... 'Mother, your son has a problem that is called, which is a beautiful name, but nobody will ever want to have it, it's called Schizophrenia' [...] (M6)*  *No, he [doctor] never clarified it to me, he never told me, in another city they did several tests, nor did the specialist from that place told me, no one ever spoke. [...] Neither there, nor here, nor anyone [...] (M3).(Bellini et al. 2016)*  *Yes, I didn’t want to accept my son’s condition. I still can’t accept it. I mean, he’s the only boy in the family. How can I accept it? (Pine – Father, 65)*  *When the doctor told us that he has schizophrenia the world crash about our ears. What are we going to do now? A chill ran down my back! We were very miserable. I wonder if there’s someone else like me in Turkey? That ia to say, you think both about the child and about yourself. How am I supposed to spend time with him? How will I do? (Juniper – Father, 60)(Akgül Gök and Duyan 2020)*    *Participant #1: “My wife and I could do nothing but cry day and night. My son used to be such a smart, tall, and handsome boy. Now he was gone. Sometimes I even want to commit suicide by jumping from upstairs, which may help end all the misery.”(Bai et al. 2020)*  *After my son was diagnosed, I was so sad. There was a blank in my head. I didn’t know what to do. I often cry secretly one by one. (C1, mother)*  *As the pillar of the family, he suddenly got such disease, what should this family do in the future! (crying). (C14, wife)(Liu and Zhang 2020)*  *“At first, I thought he was upset, maybe sad. But it never occurred to me that he might have schizophrenia until the doctor confirmed the diagnosis. It surprised the entire family as to how he got the condition.” (C3)*  *“I had to take on the role of family caregiver without being ready, as I didn’t know what works well in the family and what doesn’t, and no one else was available to assist me.” (C8)(Pan et al. 2024)*  *"Once you will determine the difficulty. You will learn what to do... First, you diagnose, what is the disease?... Then how can I overcome this difficulty? You look for a way to solve them"(Akgül Gök and Duyan 2020).*  *… She was a very good student. Her friends lead their own life. But my daughter still lives with us. She is not married; I would have grandchildren by now. Sadly, I did not do enough to help her [Father of a woman with schizophrenia_IDI_01].(Fekadu et al. 2024)* |
| **Family commitment to care** | *Commitment to care quotes*  *“I had to take on the role of family caregiver without being ready, as I didn’t know what works well in the family and what doesn’t.”(Pan et al. 2024)*  *“We spent the night without sleep too… how can I work? I was sitting and waiting him day and night” (Female-Caregiver ID13)(Demissie et al. 2021).*  *“How could I give up? He is my son. I have a duty to take care of him” (C7, mother)(Liu et al. 2022).*  *“She’s my sister! It is normal for family members to help each other when they are ill” (C16, sister)(Liu et al. 2022)*  *“Whenever I fall into despair, my elder sister does not; she is there to get me into shape. If she falls into despair, I get her into shape. There is a unity in the whole family” (Caregiver 2)(Sari and Duman 2025).*  *“I’m not staying with my sister, but my parents are there with her… I call once in two days, inquire about her condition, and ensure medication intake… Every weekend, I visit my village and spend some quality time with family… If any emergency comes up, I take leave and go there” (Participant 11, an elder brother, 28 years)(Amaresha et al. 2019)*  *It is a situation that requires high self-sacrifice . . . Without me, there is no one else to make this sacrifice . . . (65 years, ♀K7)(Bademli and Lök 2020)*  *. “I too find it too difficult to get transportation at times. Last time when he was sick and I had to get him admitted to the hospital, transportation was a big problem” (Caregiver 2, caring for a 36-year-old brother with bipolar disorder).(Mathew and Mathias 2024)*  *“The children say I should ignore him. Can you ignore him? I can’t ignore him. If you worry me and I die, none of your siblings will say you [Moses] are mad so he will look after you. Is he not alive?*  *When the person is roaming about and they know the actual family the person is coming from, when you are passing by they will be looking at you and be saying all sorts of things [….] people will be talking against you, maybe you are not trying your best.(Read and Nyame 2019)*  *“I have to do everything for my son. He cannot do anything on his own. I have to supervise on everything from eating to taking medicine” (participant no. 1). Another family caregiver asserted that: “I have to take her medication on time and not let him go out to do evil. I always must protect him from unhealthy sexual behaviors, drug abuse, substance abuse, and alcohol drinking” (participant no. 3)(Tamizi et al. 2020)*  *K. 1.19. “Well, we want our relatives to be able to provide support, don’t just ignore it, besides being ignorant, avoid it too.”*  *In addition, participant 2 said that the family wants the community to empathize with what is felt by families who have schizophrenic family members. Here’s the statement:*  *K.2.12 “Yes, they should be able to feel what we are feeling right now, don’t just talk behind our backs.”(Fauziah et al. 2024)*  *“I told my cousins: “My sister is your responsibility, my husband is a stranger and is currently ill and disabled, and so he cannot do it. Pay to take her to the hospital. After all, the ceiling of her room was also damaged and part of it completely collapsed. If everyone pays, we can hire a room in the village for her and my mom.” (Pn.1) Another participant said: “The wife’s family cannot admit the patient to a psychiatric hospital without the permission of the patient’s paternal family. If the wife’s family does it without permission, her husband’s family can sue. Because the paternal family and heirs are considered the guardians of the patient unless they decline that role.” (Pn.7)(Heydarikhayat et al. 2025)*  *children generally mentioned the lack of care from their parent with mental health issues and added that they also received little care or interest from the healthy parent who undertakes the care of their spouse and therefore is tired and fails to care for their children.(Karaca et al. 2024)*  *‘… but I believe that this is a process, and we can overcome this; we all believe this. Whenever I fall into despair, my elder sister does not; she is there to get me into shape. If she falls into despair, I get her into shape. There is a unity in the whole family, related to this disease’. (Caregiver 2)(Sari and Duman 2025)*  *I took care of him [son] all my life, It´s me who buys clothes, I give food, medicine, shoes, everything it is me, I've been fighting for him for 15 years. [...] So, I feel that I have to do everything, I am responsible for everything [...] (M2).(Bellini et al. 2016)*  *“If he got sick, I look after him and caring for him is my responsibility, I couldn’t do anything and I also sit with him. We spent the night without sleep too but he is alert in the next day also so how can I work? I was sitting and waiting him day and night …” 35-year, Female-Caregiver ID13, Sodo-district(Demissie et al. 2021)* |
| **The unrelenting burden of caregiving** | *Participant #1: “My wife and I could do nothing but cry day and night. My son used to be such a smart, tall, and handsome boy. Now he was gone. Sometimes I even want to commit suicide by jumping from upstairs, which may help end all the misery.”(Bai et al. 2020)*  *“It was a tragedy to watch my children growing without their mother. I had to take her role in addition to taking care of her… A simple example is when my daughters reach puberty.” (PC16).*  *“I did not go anywhere for three months. Even for important occasions.” (PC7).(Al-Sawafi et al. 2021)*  *My father and mother are divorced [long pause], I am living with my mother and the other two are living with my father [he is married to another woman]. My mother was selling Tela and Areki [local liquors]. I frequently dropped out of school, I was a good student but I could not proceed. She had frequent admissions. My mother works when she is fine, but now I am here with her (daughter of a woman with bipolar disorder _IDI_12).(Fekadu et al. 2024)*  *“My husband is unable to show affection as I expected. We don’t have emotional relationships like other couples.” (I 11)*  *“He always bothers me. Most of the time he is angry or not in a stable mental state. I can’t communicate with him. I prefer just to give him the medicine, take him to the doctor and perform his personal tasks.”(I 7)(Rahmani et al. 2018)*  *My illness makes me a very weak farmer which affects my family … they spent a lot in my treatment, they sold many sheep and goats… [a man with bipolar disorder_IDI_23].*  *… Previously we used to have two oxen and milk cows, but after his illness we pay for treatment and other related costs and now we do not have those… Before his illness, the land was processed early, and it gives a superior product. But after his illness, the land was given to other people because he could not work it, so we get half of it. All this creates a huge economic crisis… [son of a man with MDD_IDI_05]. (Fekadu et al. 2024)*  *“I had two younger sisters, and when my mom got sick, I became their mother and devoted everything I had earned to them. I had to care for and nurture them in place of my mother. I cleaned, fed, dressed, did laundry, and even handled school-related duties for my sisters.” (Pn.12) Another participant said: “Our place has changed. My dad was unable to work, which meant he couldn’t provide for the family. My brother left high school and became a laborer, while I sewed to help the family.” (Pn.13)*  *“Once he got sick, my life transformed completely. I always went to the gym. I used to exercise, but now I need to look after my younger brother. I seldom hang out with friends.” (Pn.4) (Heydarikhayat et al. 2025)*  *“I don’t like to go out. I don’t feel like doing anything or even go shopping because I’ve lost my morale. I used to study a lot, eat and care for myself, but I don’t have a reason to do them now. I’m always busy. Busy with chores, children’s work, problems, illness, costs; each of these makes me lose my motivation. Sometimes, I perceive myself like a butterfly who has been trapped in a spider’s web. I’m helpless in my life.*  *I’m trapped between my different roles in my life. What can I do? Which one should I put in priority? I do not know.”(Rahmani et al. 2018)*  *▪ ‘Earlier I used to do farming and look for her (care recipient) …we used to go to the farms together, but now I don’t work. All my time is used up for my daughter’. (60 years, father 2)*  *▪ ‘My routine has changed from the day he (care recipient) got sick. All my energies have been diverted. Now I either sit idle or do odd jobs. But I wonder if I have to do something in life (professionally), then who will care for him? What will happen?’ (48 years, father 3) (Soni et al. 2024)*  *“I have to look after her for 24 h. I can never leave his alone. If I get out of my house, my life will be on fire, the samovar will be on fire, the stove will be on fire, so I can’t get out of the house and have to be there for 24 h” (participant no. 1).(Tamizi et al. 2020)*  *"He would pick fights at school, pick fights at home. Anything in the house, he would break. They would smash all the glasses and everything we have." (P6)(Hasanpour et al. 2024)*  *When friends talk about such diseases or when they say crazy, of course, I don’t defend immediately. This hurts me a lot. I mean, when they say crazy, this word really hurts me a lot. Actually, they don’t say anything about my son. But I take it personally and attribute to my son even if it is said for someone else. So, I think that I should not be among these people. All these starts to seem to me nonsense. (Daphne – Mother, 55)(Akgül Gök and Duyan 2020)*  *“I kept my husband’s illness a secret from others since a lot of people ridicule those with mental health issues. Individuals often harbor negative perceptions of mental illness, which is why we prefer that only our family be aware.” (Pn.6)*  *“Our interactions with neighbors and acquaintances have decreased, and I almost never go to their homes anymore. I don’t want people to know that his behavior has changed because of his mental illness.” (Pn.15)*  *“I have a sister who is married. When she came home and found out that we had hospitalized him, she complained and asked me why did you take him to the “mad ward”? You risked the family reputation. Everyone will make fun of us tomorrow.” (Pn.4) Another participant said: “They told us if you send her to the hospital, the neighbors will say that she’s crazy and no one will want to marry her”. (Pn.15)*  *(Heydarikhayat et al. 2025)*  *I’m worried that the illness of my wife will affect my son’s future marriage. (C5, husband)(Liu and Zhang 2020)*  *My brother never accompanied me to the doctor because he feels if he goes with me to the clinic, he would also get that label of being mad. (Never-married, unemployed female, 33)(Paul and Nadkarni 2017)*  *“One of my brothers has a doctorate in economics, and he’s a very good boy in every aspect, but so far he has gone to several places to propose marriage, and as soon as they find out that our mother is ill, they immediately reject him. Why shouldn’t my brother have a good marriage with this situation?”*  *“If you want to get a wife for your child, people say they shouldn’t give him a wife; one of their children is in the mental hospital. I heard with my own ears that they say ‘they are all crazy’.”(Latifian et al. 2024)*  *“If someone wants to propose my daughters, if they know that their father has this problem, they regret. I have two daughters, one of them studied Law and another one studied Mechanics. Actually when the suitors got that he is ill, they say if we get married with them, perhaps, we will have to support our wife’s family.(Kargar et al. 2021b)*  *“Last time, my cattle entered another person’s farm and were grazing there; because of that, the owner of the farm was hitting my cattle. My son [person with BD] asked him why he hit the cattle and he [owner of the farm] tried to hit him with an axe but he ran away and escaped. If the cattle were belonging to another person, they may not have tried to hit that person.” 63-year, Male-Caregiver ID012, Sodo-district. (Demissie et al. 2021)*  *“On my son’s wedding, I was sleeping when she attacked me, and my face was full of blood. People were asking, but I lied, and it was embarrassing.” (PC4). (Al-Sawafi et al. 2021)*  *I have sleepless nights. When she is awake, I can’t sleep and her illness has affected my health. It gives me ill health and all of this has worsened my high blood pressure. I had hypertension before her illness started but it was not this bad, it’s getting worse. I’m taking treatment, too. (Interview 5.)*  *How can I be well with this type of sickness? I cannot sleep. Look at me. I am so emaciated because of my daughter’s illness. It makes me think all the time. I was not like this before...My daughter’s illness is really worrying me. I’ve left my trade and everything. Financially, it has ruined my life. (Interview 11.) (Jack-Ide et al. 2013).*  *Since I don’t have time for myself most of the time, I can get angry at even the smallest thing. In such cases, I usually take my anger out on myself or my relatives. (55 years, Father 1)*  *My relatives and brothers look at my child as crazy. The feeling of not being able to stand behind my child makes me angry. I am very angry with myself. (48 years, Father 4)*  *Because of my child, I get angry at even the smallest things and I get very depressed. But I’m used to it now. I am not 50 years old. I am 70 years old. How long will I live that is my test. (70 years, Mother 3)(Kalayci et al. 2023).*  *‘Now I am always thinking too much and thinking for how long I will carry on looking after an adult. This is really eating me inside; you can’t be happy as a family when someone is not feeling well within the family.’ (IDI, mother, age 60) (Marimbe et al. 2016).*  *“I'm tired of saying, 'Please take a bath'. I just told her to take a shower again and again. My neighbour said, 'Cut your hair; I'll give you 200', but she didn't want to do it." (K16) "Yeah, you're already too old—not a six-year-old kid—and still asking me to feed you. How would I let it be?*  *“she only wants me to keep accompanying her; but how do I treat her well if she keeps asking me to accompany her? (K15)”(Wulandari et al. 2024) .*  *“That was my destiny when I was off work and when I was resting; that was the job” (dad, family 7)(Bedoya Hernandez and Builes Correa 2013).*  *ʻSometimes we have a strong feeling of helplessness. We have some problems when he (the patient) is at home, and other problems when he is in hospital. There is no peace in our life. There is always commotion in the house. All of us like to escape from this situation. At this age, I’m getting depressed and not inclined to do anythingʼ. (F7) (Lohrasbi et al. 2024).*  *▪ ‘I could not afford the medicine; I have spent a lot on transportation as he has to be evaluated by the doctor every 15 days’. (60 years, father 2)*  *▪ ‘Sometimes, there is no money. Before coming to this public facility, I was taking him to a private doctor, the treatment cost around rupees four-five thousand per month …how do I arrange that much, I can’t!’ (50 years, mother 5)*  *▪ ‘Many times, I have to borrow money. The medicines are expensive.’(65 years, mother 7) (Soni et al. 2024)*  *I (caregiver) am worried, about what will happen to him (care recipient) after me, who will take care of him (care recipient). I (the caregiver) am unclear about his future care in case something uneventful happens to me (caregiver). I just wish either God takes him away or frees him from this illness. (65 years, mother 1)*  *▪ ‘She (care recipient) worries, ‘as long as my parents are there, they are taking care of me (patient), who will care for me, after them.’ … It will be better only for her to die before me, otherwise, no one will take care of her. (60 years, father 2)*  *▪ ‘I will take care of her (care recipient) children but I wish God either fix it or take her away’. (40 years, sister 6)(Soni et al. 2024)*  *“If I knew that he was sick, I wouldn’t have married him," one wife stated bluntly. "If I knew, I would not have fallen myself into this disaster” (ICG 10)(Kargar et al. 2021a).*  *“Sometimes I have decided to divorce. But I have not done it for my kids. I can’t tolerate him anymore” (ICG 11)(Kargar et al. 2021a).*  *“I am thinking of leaving the house, that’s it, I even need divorce… my head gets hot, that’s it, I am also sick” (FGD 02 participant 2, female caregiver)(Asher et al. 2017).*  *“… The families are tired too. They intend to leave the patient; they are tired of the patient and the treatment process as well” (N3)(Lohrasbi et al. 2023a).*  *‘Sometimes I truly hate him…I am in a bad mood, and I truly hate him every time he scolds me. What I said is, “If it were not restricted by law, I truly would like to kill you”. (Peng et al. 2022).*  *“God knows how hard it is to live with these patients. I wish they could be given an air injection and we could get rid of them” (F15)(Lohrasbi et al. 2023a).*  *“I just wish either God takes him away or frees him from this illness” (65 years, mother 1)(Soni et al. 2024).*  *“This year I pray to take one of us (either my mother or me), I do not want to live such life… once I try to harm myself, but God saved me” [Daughter of a woman with bipolar disorder_IDI_12](Fekadu et al. 2024).*  *“what will happen to him (care recipient) after me?” (65 years, mother 1)(Soni et al. 2024).* |
| **Forging resilience: strategies of enduring care** | *“Schizophrenia is a serious mental illness requiring long-term care. Accepting it will reduce a lot of anger and depression in my life” (C3)(Pan et al. 2024).*  *“Firstly, when hearing the diagnosis of my sister, I accept that my sister has mental problem. After that I tried to find ways to deal with problems. I also tried to see the positive dimension of the problem; strengthening myself by telling myself this problem is not only mine and there are people living in a bad condition which might be very difficult as compared to mine.” (Abebe)(Hailegabriel and Berhanu 2023)*  *“When I think of her inability to marry, have children, or work like others in the future, I feel sad and angry. It’s hard for me to admit she is ill. But as her mother… I had to accept the reality.” (C10)(Pan et al. 2024)*  *“Once you will determine the difficulty. You will learn what to do to overcome this difficulty. For example, as a doctor do you treat a patient without a diagnosis? First, you diagnose, what is the disease? Then you treat it... What is this difficulty resulted from? Then how can I overcome this difficulty? You look for a way to solve them.” (Olive – Father, 66)(Akgül Gök and Duyan 2020).*  *“..Yes, the treatment is only relieved, we have to be patient with God, surrender..” (P1)*  *“..Have you ever talked to a spiritual teacher? the best vent is already with people with Allah..” (P2)*  *“..I'm just praying, we're asking God for it, I hope I'll get better and not what about me..” (P3) (Gumilang 2023)*  *“Whenever the aggressive behaviour of my son begins, I immediately enter into my room, lock the door, kneel down and pray for Almighty God” (50 years old mother)(Iseselo et al. 2016).*  *Another participant framed it simply: “I'm just praying, we're asking God for it, I hope I'll get better” (P3)(Gumilang 2023).*  *“You need to accept, no way, that is your child… where would you ask help? That is your gift from Almighty God, you have to accept” (58 years old father)(Iseselo et al. 2016).*  *“It is merely my faith which makes me to be here.”(Iseselo et al. 2016)*  *“A person can only get better to a certain point, and coming to terms with the fact they won’t be cured can provide some comfort” (C2)(Pan et al. 2024).*  *In some cultural contexts, this was framed as tamjai (reconciliation to a situation), a conscious adjustment of one's mindset to the reality of lifelong caregiving, guided by the philosophy that “Whatever happens, happens.”(Kanungpiarn et al. 2021)*  *“I always try to talk to somebody, but they don’t understand me. There is no one who understands me, there are attendants in the hospital, it makes me feel good to talk to them Because we understand each other and support each other.” (20 years, ♂, K5)(Bademli and Lök 2020)*  *“I got more experience of care from other caregivers, which helped me a lot” (C3, mother)(Liu and Zhang 2020).*  *“They told me about their care experience, which touched me and increased my courage and confidence” (C7, mother)(Liu and Zhang 2020).*  *“My mother requires substantial support and care, yet my child also necessitates companionship. I take my child out to play once a week. This gives me a short break to relax and rest” (C2)(Pan et al. 2024).*  *My husband cannot help me take care of our son. He always blames me, saying I’m not doing a good enough job caring for him. It’s tough to know what to do in those situations, you know? (C16)(Pan et al. 2024)*  *‘Who else will do it? His father came here only once, he didn’t come again. The whole problem is carried by me.’ As she put it: ‘I have no man except God’. She complained that Moses’ father had contributed only a small sum to meet the substantial fees demanded by the shrine.(Read and Nyame 2019)*  *Inviting patients to do joint and social activities is supported by the category of involving patients in daily activities.…*  *I told him to sit there with me so that we can talk together with a friend. If he’s alone, he’ll be lost in thought… (Participants 1, 3, and 5).…I send you to the stall, for example, to shop…to the prayer room to pray (Participants 2, 4, and 7)(Budiarto and Mustikasari 2024)*  *"…I taught him, speak well, call the name of the person you are talking to, smile, don't get angry or yell, just talk to neighbors as needed…" (P10 and P17).(Fitryasari et al. 2018)*  *“Once he takes his medicine and starts to feel better, his brothers take him to his job. If my brother or father had something to do, they would bring him along as well. My husband used to be a builder. However, he can no longer perform the tasks he used to at the heights, he only engages in simpler activities.” (Pn.8)(Heydarikhayat et al. 2025)*  *‘I have a dog; my dog is very loyal to me because I take her out and walked her around all the time. We always spend time together; she sleeps in my room. So, my family is the most valuable thing that keeps me alive’. (Patient 8)*  *‘Thanks to our dog, she helped my daughter develop social activities because she wanted to play games, my daughter wants to sleep, but she goes and puts her head down on her etc… She increased the love. It was good for us too’.*  *‘We somehow thought about what she does well and realized that she can make very good coffee. I said, ‘Oh, you make the best coffee in this house’. We talked with her aunt and her sister, together with the father. They said, Oh, we've never had such good coffee. Now she makes coffee 3–4 times a day’. (Caregiver 8) (Sari and Duman 2025)*  *“So I teach him/her to make a doormat” (P4)*  *“Now she is only sweeping the floor. She loves to sweep the floor” (P5)*  *“….later he will talk to welcome guests, friends, relatives …” (P6)(Yunita et al. 2020)* |
| **Voiced needs and priorities** | *ʻMany of them have no awareness of the treatment process. Despite the fact that they involve in the treatment of their patient, they don’t know that the treatment is a long process; they have no information about the nature of the illnessʼ (A psychiatrist nurse). (N9)*  *“We would like to know why this illness appeared all of a sudden. My child was absolutely all right before few months! Why it happened?” (Kumar et al. 2019)*  *“We need to know some information such as what is the nature of this illness? Why does it occur? Why is my brother behaving like this?” (Participant 3)(Amaresha et al. 2015).*  *“Many of them have no awareness of the treatment process… they have no information about the nature of the illness” (N9)(Lohrasbi et al. 2023b).*  *“I don’t know the side effects of the tablets… If he [brother] stops medication we don’t know what problems will arise… I need to know about recognizing early warning signs. It will help me to prevent further worsening of the illness.” (Participant 5)*  *‘‘I am often involved in a verbal exchange with my brother. . . I am frustrated with his behaviour. He doesn’t listen to me. . .. I know he has a problem, but I cannot control myself. . .. Feel irritated. . .. I need some tips in talking with my brother patiently.’’ (Participant 4, 34 years, Elder brother)*  *‘‘These days I am having lot of pressure both personally and professionally. I feel restless. . .Not able to spend time with my ﬁance´e and friends. Mother calls me and complains about my sister. All these things disturb me a lot. Sometimes I feel stressed out and shout at others for minor reasons. . .I really want to learn how to manage this.’’ (Participant 11, 28 years, Elder brother)*  *‘‘He [brother] and one of my maternal relatives have the same illness. Sometimes I am a bit worried about this. . .I need to talk with you people [professionals] and get clariﬁcation about that [heredity]’’ (27 years, Younger brother)(Amaresha et al. 2015)*  *“Sometimes, I don't know how to behave. I would like to learn this most” (P5)(Özgönül and Bademli 2022),*  *“I know he has a problem, but I cannot control myself… I need some tips in talking with my brother patiently” (Participant 4). “My parents think that if she gets married, the issues will be resolved… If I get information, I will educate them at home” (Participant 11))(Amaresha et al. 2015).*  *“Written information is also helpful… it helped me a lot; [now] we know that if [things] go this way, we do it this way” (FGD, brother, age 36)(Marimbe et al. 2016).*  *"I gotta be honest; I don't think the treatment personnel can really help us at all. I mean, what do they even do in that department? Can they actually do something to help me? If they could, I wouldn't be feeling so darn alone right now."(Asgari et al. 2023)*  *“Sometimes, I don't know how to behave. I would like to learn this most.” (P5) “Nobody gives us information about the illness. I try to get information from related books.”(Özgönül and Bademli 2022)*  *“It would be great if the family could also have a say in the therapeutic procedures, and family members should be involved in the treatments and therapeutic efforts. Why haven't you informed us about the therapeutic methods or efforts that have been or will be used for my brother until now? It's our absolute right as my mother and I to be part of these discussions and decisions.”(Asgari et al. 2023)*  *“Sometimes they can ignore the family. However, the family has great importance in this process. Because the family is always together with the patient. Most of the time, it seems like the patient and family are unimportant for the doctors.”(Akgül Gök and Duyan 2020)*  *“What annoyed me so much was the fact that they wanted us to treat our mother. For example, they wanted us to talk to her and talk to her in that way A child does not understand this issue.... Do not use a child as a tool to take a mother to a physician. The physician should not use the child and ask him/her to tell lies.”(Zarei et al. 2021)*  *“Well, we want our relatives to be able to provide support, don’t just ignore it, besides being ignorant, avoid it too” (K. 1.19). “feel what we are feeling right now, don’t just talk behind our backs” (K.2.12)(Fauziah et al. 2024).*    *‘… I think it would be very interesting to be in a group of family members or similarly a chat with family members […] I think maybe they could help me or maybe I could help them through my experiences and knowing what has happened […] Yes, I think it would be useful because now that I have started psychotherapy, I see that talking about it helps a lot […] so talking about this with people who have shared this problem could help me, I think, and I think I could help others …’ (Son of Mrs. C) (Vargas-Huicochea et al. 2018).*  *“… Having a group or a space, especially to know that you aren’t the only one or to see the experiences of others. I feel like it would help a lot to see yourself in reflected in others…”(Father of Mr. A)(Vargas-Huicochea et al. 2018).*  *“If they do support groups like the one for people living with AIDS, it will be fine. Those people with HIV support each other, so we can also support each other.”(FDG, mother, age 61)(Marimbe et al. 2016).*  *‘‘Sometimes, I accompany my sister to the hospital for follow ups. If there is an opportunity, I would be interested to meet other families who have patients with similar illness. I want to understand their struggles and learn how they are caring for their relatives.’’ (Participant 11, 28 years, Elder brother)(Amaresha et al. 2015)*  *“If we get such group which can help to socialize and assist such children and intermingle with those who are mentally stable, could greatly help them. …….because we toil to go here and there as a result we become weary in vain.” (A 42 years old father).*  *“You become tired of everything; you wish to have new ideas or people to help or group to mix with for getting new challenges……..you find that we are also tired of thinking, tired of strength, at the same time running short of money.” (A 40 years old mother).(Iseselo et al. 2016)*  *“Things that will be done to fight against stigmatization should reach more people and be increased.”(Oz et al. 2022)*  *“My daughter has marital disagreement, and her husband asked [for] legal separation from the court. Since I am the only caregiver for my daughter, I couldn’t even attend the appointment of the court… I wish to have somebody who has knowledge about legal terms and stand on behalf of her.”(Hailegabriel and Berhanu 2023)*  *“We fight for money. Well, I'm looking for money. I go to school. I need money. My father cannot work, and my mother has to work. I should do something as well.”(Zarei et al. 2021)*  *“My daughter has never worked before. Our whole family’s income depends on my husband’s retirement pension… the treatment fee for the patient is expensive” (C3)(Chen et al. 2019).*  *“We cannot afford medications... If we get it for free then we can arrange his travel expenditure to come for follow-ups” (Participant 3)(Amaresha et al. 2015).*  *“The free medications alleviated our financial burden for a large extent, but 99% of them were Generation 1 with obvious side effects. We hope that the free medication directory can extend to Generation 2 medications with fewer side-effects.” (C1)(Chen et al. 2019)* |

**References**

**Akgül Gök F and Duyan V** (2020) 'I wanted my child dead' – Physical, social, cognitive, emotional and spiritual life stories of Turkish parents who give care to their children with schizophrenia: A qualitative analysis based on empowerment approach. *INTERNATIONAL JOURNAL OF SOCIAL PSYCHIATRY* **66**(3)**,** 249–258. <https://doi.org/10.1177/0020764019899978>.

**Al-Sawafi A, Lovell K, Renwick L and Husain N** (2021) Exploring the experience of relatives living with individuals diagnosed with schizophrenia in Oman: A qualitative study. *Journal of Psychiatric and Mental Health Nursing* **28**(6)**,** 1029–1040. <https://doi.org/doi:https://dx.doi.org/10.1111/jpm.12786>.

**Amaresha AC, Joseph B, Agarwal SM, Narayanaswamy JC, Venkatasubramanian G, Muralidhar D and Subbakrishna DK** (2015) Assessing the needs of siblings of persons with schizophrenia: A qualitative study from India. *Asian Journal of Psychiatry* **17,** 16–23. <https://doi.org/doi:https://dx.doi.org/10.1016/j.ajp.2015.07.011>.

**Amaresha AC, Venkatasubramanian G and Muralidhar D** (2019) Perspectives about Illness, Attitudes, and Caregiving Experiences among Siblings of Persons with Schizophrenia: A Qualitative Analysis. *Indian J Psychol Med* **41**(5)**,** 413–419. <https://doi.org/doi:10.4103/ijpsym.Ijpsym_318_19>.

**Asgari M, Adib M, Nayeri ND and Rezayat F** (2023) Family caregivers' perspectives on barriers to caring for patients with schizophrenia: A descriptive qualitative study. *Nursing Practice Today* **10**(3)**,** 239–249. <https://doi.org/10.18502/npt.v10i3.13433>.

**Asher L, Fekadu A, Teferra S, De Silva M, Pathare S and Hanlon C** (2017) "I cry every day and night, I have my son tied in chains": physical restraint of people with schizophrenia in community settings in Ethiopia. *Globalization &amp; Health* **13,** 47–47. <https://doi.org/10.1186/s12992-017-0273-1>.

**Bademli K and Lök N** (2020) Feelings, thoughts and experiences of caregivers of patients with schizophrenia. *Int J Soc Psychiatry* **66**(5)**,** 452–459. <https://doi.org/doi:10.1177/0020764020916211>.

**Bai X-L, Luo Z-C, Wang A, Guan Z-Y, Zhong Z-Y, Sun M and Tang S-Y** (2020) Challenge of parents caring for children or adolescents with early-stage schizophrenia in China: A qualitative study. *Perspectives in Psychiatric Care* **56**(4)**,** 777–784. <https://doi.org/doi:https://dx.doi.org/10.1111/ppc.12492>.

**Bedoya Hernandez MH and Builes Correa MV** (2013) Context of the care provided to a family member with bipolar disorder in Antioquia, Colombia. *Iatreia* **26(4),** 419–429.

**Bellini LC, Soares Cunha M, Fernandes Cardoso da Silva T and Ciccone Giacon BC** (2016) THE EXPERIENCE OF BEING A MOTHER OF AND INDIVIDUAL WITH SCHIZOPHRENIA. *Ciencia, Cuidado e Saude* **15**(4)**,** 701–707. <https://doi.org/doi:10.4025/cienccuidsaude.v15i4.34527>.

**Budiarto E and Mustikasari** (2024) The Meaning of Family's Adaptation Experiences Among Schizophrenia Caregivers in the Tidal Flood Areas in Indonesia: A Phenomenology Study. *SAGE Open Nurs* **10,** 23779608241289099. <https://doi.org/doi:10.1177/23779608241289099>.

**Chen L, Zhao Y, Tang J, Jin G, Liu Y, Zhao X, Chen C and Lu X** (2019) The burden, support and needs of primary family caregivers of people experiencing schizophrenia in Beijing communities: a qualitative study. *BMC Psychiatry* **19**(1)**,** 75. <https://doi.org/doi:10.1186/s12888-019-2052-4>.

**Demissie M, Hanlon C, Ng L, Fekadu A and Mayston R** (2021) Why doesn't God say "enough"? Experiences of living with bipolar disorder in rural Ethiopia. *Social Science & Medicine* **270**. <https://doi.org/doi:https://dx.doi.org/10.1016/j.socscimed.2020.113625>.

**Fauziah S, Balqis UM, Obar O, Suryadin A and Chairunisa S** (2024) Family journey in the process of recovery of schizophrenia: A qualitative study. *Journal of Integrative Nursing* **6**(2)**,** 83–89. <https://doi.org/doi:10.4103/jin.jin_32_24>.

**Fekadu W, Craig TKJ, Hanlon C, Mayston R and Fekadu A** (2024) Distributed impact of severe mental health conditions in rural Ethiopia, a qualitative study. *BMC Psychiatry* **24**(1)**,** 670. <https://doi.org/doi:10.1186/s12888-024-06124-0>.

**Fitryasari R, Yusuf A, Nursalam, Tristiana RD and Nihayati HE** (2018) Family members' perspective of family Resilience's risk factors in taking care of schizophrenia patients. *Int J Nurs Sci* **5**(3)**,** 255–261. <https://doi.org/doi:10.1016/j.ijnss.2018.06.002>.

**Gumilang R** (2023) CHRONIC SORROW FAMILY CAREGIVER OF CLIENTS WITH SCHIZOPHRENIA IN WEST BANDUNG REGENCY. *Indonesian Nursing Journal of Education &amp; Clinic (INJEC)* **8**(1)**,** 47–57. <https://doi.org/doi:10.24990/injec.v8i1.535>.

**Hailegabriel K and Berhanu Z** (2023) Caregiving challenges and coping strategies of family caregivers for relatives diagnosed with bipolar disorder in Ethiopia. *Families in Society* **104**(1)**,** 5–19. <https://doi.org/doi:https://dx.doi.org/10.1177/10443894221129325>.

**Hasanpour M, Poortaghi S, Zare Z, Yahyavi ST and Shahsavari H** (2024) The Voice of the Family of Schizophrenic Patients in the Continuum of Patient Care: A Qualitative Study. *Iranian Journal of Psychiatry and Behavioral Sciences* **18**. <https://doi.org/doi>:.

**Heydarikhayat N, Darban F and Farokhzadian J** (2025) Putting the broken pieces of life together, Baloch family caregivers' confrontation with schizophrenia: a phenomenological study. *BMC Psychol* **13**(1)**,** 87. <https://doi.org/doi:10.1186/s40359-025-02358-z>.

**Iseselo MK, Kajula L and Yahya-Malima KI** (2016) The psychosocial problems of families caring for relatives with mental illnesses and their coping strategies: A qualitative urban based study in Dar es Salaam, Tanzania. *BMC Psychiatry* **16**. <https://doi.org/doi>:.

**Jack-Ide IO, Uys LR and Middleton LE** (2013) Caregiving experiences of families of persons with serious mental health problems in the Niger Delta region of Nigeria. *Int J Ment Health Nurs* **22**(2)**,** 170–179. <https://doi.org/doi:10.1111/j.1447-0349.2012.00853.x>.

**Kalayci E, Uzunaslan İ and Uzunaslan Ş** (2023) Caregiver burden experiences of caregivers of patients with schizophrenia: A qualitative inquiry. *Int J Soc Psychiatry* **69**(3)**,** 543–550. <https://doi.org/doi:10.1177/00207640221114564>.

**Kanungpiarn T, Tungpunkom P, Kantaruksa K and Chaloumsuk N** (2021) Hopeful Endless Caring to Maintain Normal Life: A Grounded Theory of Thai Mothers' Caring for Adult Children with Schizophrenia. *Pacific Rim International Journal of Nursing Research* **25**(2)**,** 171–184.

**Karaca A, Şener DK and Kundakçi N** (2024) Being a child of a parent with a diagnosis of schizophrenia or bipolar disorder: A qualitative study. *Arch Psychiatr Nurs* **50,** 94–99. <https://doi.org/doi:10.1016/j.apnu.2024.03.011>.

**Kargar M, Faghihi S and Nazari M** (2021a) Care burden dimensions of informal caregivers having patients with bipolar disorder (challenges and alternatives) (qualitative study). *Journal of Education and Health Promotion* **10**(1). <https://doi.org/10.4103/jehp.jehp_943_20>.

**Kargar M, Faghihi SA and Nazari M** (2021b) Care burden dimensions of informal caregivers having patients with bipolar disorder (challenges and alternatives) (qualitative study). *J Educ Health Promot* **10,** 282. <https://doi.org/doi:10.4103/jehp.jehp_943_20>.

**Khadem H, Shahidi S, Zarani F and Panaghi L** (2022) An Uncertain Impairing Traumatic Relationship within the Circle of Rejection, Anger, and Freedom: An Interpretive Phenomenological Analysis of the Subjective Burden of Caregivers of Patients with Bipolar Disorder. *International Journal of Behavioral Sciences* **16**(2)**,** 125–133. <https://doi.org/doi:10.30491/IJBS.2022.336211.1783>.

**Kumar G, Sood M, Verma R, Mahapatra A and Chadda RK** (2019) Family caregivers' needs of young patients with first episode psychosis: A qualitative study. *International Journal of Social Psychiatry* **65**(5)**,** 435–442. <https://doi.org/doi:https://dx.doi.org/10.1177/0020764019852650>.

**Latifian M, Abdi K, Raheb G, Islam SMS and Alikhani R** (2024) The experiences of bipolar patients' families regarding stigma coping strategies in Tehran: A qualitative study. *Current Psychology: A Journal for Diverse Perspectives on Diverse Psychological Issues* **43**(5)**,** 4179–4190. <https://doi.org/doi:https://dx.doi.org/10.1007/s12144-023-04620-2>.

**Liu N and Zhang J** (2020) Experiences of caregivers of family member with schizophrenia in China: A qualitative study. *Perspectives in Psychiatric Care* **56**(1)**,** 201–212. <https://doi.org/doi:https://dx.doi.org/10.1111/ppc.12412>.

**Liu SH, Hsiao FH, Chen SC, Shiau SJ and Hsieh MH** (2022) The experiences of family resilience from the view of the adult children of parents with bipolar disorder in Chinese society. *J Adv Nurs* **78**(1)**,** 176–186. <https://doi.org/doi:10.1111/jan.15008>.

**Lohrasbi F, Maghsoudi J, Alavi M and Akbar M** (2023a) Care Bermuda, families of the patients with chronic mental disorders in Iran surrounded by psychosocial problems and needs: a qualitative study. *Annals of Medicine and Surgery* **86(6),** 3357–3366.

**Lohrasbi F, Maghsoudi J, Alavi M and Akbar M** (2023b) Care Bermuda, families of the patients with chronic mental disorders in Iran surrounded by psychosocial problems and needs: a qualitative study. *Annals of Medicine and Surgery* **86,** 3357–3366. <https://doi.org/doi>:.

**Lohrasbi F, Maghsoudi J, Alavi M and Akbar M** (2024) Care Bermuda, families of the patients with chronic mental disorders in Iran surrounded by psychosocial problems and needs: a qualitative study. *Ann Med Surg (Lond)* **86**(6)**,** 3357–3366. <https://doi.org/10.1097/ms9.0000000000000626>.

**Marimbe BD, Cowan F, Kajawu L, Muchirahondo F and Lund C** (2016) Perceived burden of care and reported coping strategies and needs for family caregivers of people with mental disorders in Zimbabwe. *Afr J Disabil* **5**(1)**,** 209. <https://doi.org/doi:10.4102/ajod.v5i1.209>.

**Mathew SC and Mathias T** (2024) Unveiling the Unseen: Exploring the Lived Experiences of Family Caregivers of Young Adults with Chronic Mental Disorders: A Qualitative Phenomenological Study. *Journal of Clinical and Diagnostic Research* **18,** LC20–LC26. <https://doi.org/doi>:.

**Olwit C, Musisi S, Leshabari S and Sany I** (2015) Chronic Sorrow: Lived Experiences of Caregivers of Patients Diagnosed With Schizophrenia in Butabika Mental Hospital, Kampala, Uganda. *Archives of Psychiatric Nursing* **29**(1)**,** 43–48. <https://doi.org/10.1016/j.apnu.2014.09.007>.

**Oz YC, Duran S and Incedere A** (2022) Requirements of caregivers of patients with schizophrenia: A qualitative study in Turkey. *Archives of Psychiatric Nursing* **41,** 81–86. <https://doi.org/doi:https://dx.doi.org/10.1016/j.apnu.2022.07.023>.

**Özgönül ML and Bademli K** (2022) Ethical problems experienced by family caregivers of patient with schizophrenia: A hermeneutic phenomenological study. *Perspect Psychiatr Care* **58**(4)**,** 2127–2136. <https://doi.org/doi:10.1111/ppc.13039>.

**Pan Z, Li T, Jin G and Lu X** (2024) Caregiving experiences of family caregivers of patients with schizophrenia in a community: a qualitative study in Beijing. *BMJ Open* **14**(4)**,** e081364. <https://doi.org/doi:10.1136/bmjopen-2023-081364>.

**Paul S and Nadkarni VV** (2017) A qualitative study on family acceptance, stigma and discrimination of persons with schizophrenia in an Indian metropolis. *International Social Work* **60**(1)**,** 84–99. <https://doi.org/doi:https://dx.doi.org/10.1177/0020872814547436>.

**Peng MM, Ma Z and Ran MS** (2022) Family caregiving and chronic illness management in schizophrenia: positive and negative aspects of caregiving. *BMC Psychol* **10**(1)**,** 83. <https://doi.org/doi:10.1186/s40359-022-00794-9>.

**Rahmani F, Ebrahimi H, Seyedfatemi N, Namdar Areshtanab H, Ranjbar F and Whitehead B** (2018) Trapped like a butterfly in a spider's web: Experiences of female spousal caregivers in the care of husbands with severe mental illness. *J Clin Nurs* **27**(7)**,** 1507–1518. <https://doi.org/doi:10.1111/jocn.14286>.

**Read UM and Nyame S** (2019) “It is left to me and my god”: Precarity, responsibility, and social change in family care for people with mental illness in Ghana. *Africa Today* **65**(3)**,** 3–27. <https://doi.org/doi:10.2979/africatoday.65.3.02>.

**Sari A and Duman ZÇ** (2025) Family resilience experiences of individuals with schizophrenia‐family caregivers dyads: Qualitative study. *Journal of Psychiatric &amp; Mental Health Nursing (John Wiley &amp; Sons, Inc.)* **32**(1)**,** 125–137. <https://doi.org/doi:10.1111/jpm.13091>.

**Soni A, Kumar R and Das A** (2024) Experiences of the family caregivers of persons with schizophrenia in north Indian region: A qualitative inquiry. *Arch Psychiatr Nurs* **51,** 17–24. <https://doi.org/doi:10.1016/j.apnu.2024.05.005>.

**Tamizi Z, Fallahi-Khoshknab M, Dalvandi A, Mohammadi-Shahboulaghi F, Mohammadi E and Bakhshi E** (2020) Caregiving burden in family caregivers of patients with schizophrenia: A qualitative study. *J Educ Health Promot* **9,** 12. <https://doi.org/doi:10.4103/jehp.jehp_356_19>.

**Vargas-Huicochea I, Berenzon S, Rascon ML and Ramos L** (2018) A bittersweet relationship: What does it mean to be the caregiver of a patient with bipolar disorder? *International Journal of Social Psychiatry* **64**(3)**,** 207–216. <https://doi.org/doi:https://dx.doi.org/10.1177/0020764018758124>.

**Verity F, Turiho A, Mutamba BB and Cappo D** (2021) Family care for persons with severe mental illness: experiences and perspectives of caregivers in Uganda. *Int J Ment Health Syst* **15**(1)**,** 48. <https://doi.org/doi:10.1186/s13033-021-00470-2>.

**Wulandari D, Keliat BA, Susanti H and Besral** (2024) Family Experiences in Caring for People with Schizophrenia: A Qualitative Study. *Malaysian Journal of Nursing (MJN)* **16**(1)**,** 124–134. <https://doi.org/10.31674/mjn.2024.v16i01.013>.

**Yunita FC, Yusuf A, Nihayati HE and Hilfida NH** (2020) Coping strategies used by families in Indonesia when caring for patients with mental disorders post-pasung, based on a case study approach. *Gen Psychiatr* **33**(1)**,** e100035. <https://doi.org/doi:10.1136/gpsych-2018-100035>.

**Zarei S, Zeighami R and Javadi AS** (2021) Needs of Children of Parents with Schizophrenia. *Preventive Care in Nursing &amp; Midwifery Journal* **11**(3)**,** 32–39. <https://doi.org/doi:10.52547/pcnm.11.3.32>.
