# Supplementary material for: Experiences, needs and priorities of family caregivers of people with severe mental health conditions in low- and middle-income countries: A systematic review of qualitative studies
Source: Glob Ment Health (Camb). 2026 May 13;13:e115. doi: 10.1017/gmh.2026.10224 (PMC13244235; doi:10.1017/gmh.2026.10224)
Supplement: Enkoyee et al. supplementary material [file S2054425126102246sup001.zip › supplementary file 1.docx]

**Search terms used**

“severe mental health condition*” OR Psychosis OR "Psychotic Disorder*" OR Schizophrenia OR "Bipolar Disorder*" OR “schizophrenia spectrum disorder*” OR “bipolar spectrum disorder*” OR "severe mental illness*" OR "serious mental illness*"

AND

"Family member*" OR Family* OR Spouse* OR mother* OR father* OR Parent* OR Sibling* OR Child* OR “family caregiver*” OR relative* OR carer* OR caregiver*

AND

experience* or perception* or attitude* or feeling* or belief* or view* or perspective* or opinion* or "lived experience*" or role* or "family relation*" or support* or "social support" or "family support" or "community support" or "peer support" or "support need*" or "help-seeking" or help* or coping or "caregiver support*" or "family support*" or "mental health*" or "mental health support*" or "financial support*" or "practical support*" or "psychological support*" or access* or "barriers to support" or burden or "caregiver burden"

AND

"Qualitative Research" OR "qualitative method*" OR "qualitative study" OR "Focus Group*" OR "Interview*" OR Narration* OR phenomenolog* OR "thematic analysis" OR "grounded theory" OR ethnograph* OR "narrative analysis" OR Observation* OR narrative* OR “lived experience*”

AND

("deprived countries" or "deprived country" or "deprived nation" or "deprived nations" or "deprived population" or "deprived populations" or "deprived world" or "developing countries" or "developing country" or "developing economies" or "developing economy" or "developing nation" or "developing nations" or "developing population" or "developing populations" or "developing world" or "lami countries" or "lami country" or "less developed countries" or "less developed country" or "less developed economies" or "less developed economy" or "less developed nation" or "less developed nations" or "less developed population" or "less developed populations" or "less developed world" or "lesser developed countries" or "lesser developed country" or "lesser developed economies" or "lesser developed economy" or "lesser developed nation" or "lesser developed nations" or "lesser developed population" or "lesser developed populations" or "lesser developed world" or "LMIC" or "LMICS" or "low gdp" or "low gnp" or "low gross domestic" or "low gross national" or "low income countries" or "low income country" or "low income economies" or "low income economy" or "low income nation" or "low income nations" or "low income population" or "low income populations" or "lower gdp" or "lower gnp" or "lower gross domestic" or "lower gross national" or "lower income countries" or "lower income country" or "lower income economies" or "lower income economy" or "lower income nation" or "lower income nations" or "lower income population" or "lower income populations" or "middle income countries" or "middle income country" or "middle income economies" or "middle income economy" or "middle income nation" or "middle income nations" or "middle income population" or "middle income populations" or "poor countries" or "poor country" or "Poor Economies" or "Poor Economy" or "poor nation" or "poor nations" or "poor population" or "poor populations" or "poor world" or "poorer countries" or "poorer country" or "Poorer Economies" or "Poorer Economy" or "poorer nation" or "poorer nations" or "poorer population" or "poorer populations" or "poorer world" or "third world" or "transitional countries" or "transitional country" or "Transitional Economies" or "Transitional Economy" or "under developed countries" or "under developed country" or "under developed economies" or "under developed economy" or "under developed nation" or "under developed nations" or "under developed population" or "under developed populations" or "under developed world" or "under served countries" or "under served country" or "under served nation" or "under served nations" or "under served population" or "under served populations" or "under served world" or "underdeveloped countries" or "underdeveloped country" or "underdeveloped economies" or "underdeveloped economy" or "underdeveloped nation" or "underdeveloped nations" or "underdeveloped population" or "underdeveloped populations" or "underdeveloped world" or "underserved countries" or "underserved country" or "underserved nation" or "underserved nations" or "underserved population" or "underserved populations" or "underserved world" or Afghanistan or Albania or Algeria or Angola or Antigua or Barbuda or Argentina or Armenia or Armenian or Aruba or Azerbaijan or Bangladesh or Benin or Byelarus or Byelorussian or Belarus or Belorussian or Belorussia or Belize or Bhutan or Bolivia or Bosnia or Herzegovina or Hercegovina or Botswana or Brazil or Bulgaria or Burkina Faso or Burkina Fasso or Upper Volta or Burundi or Urundi or Cambodia or Khmer Republic or Kampuchea or Cameroon or Cameroons or Cameron or Camerons or Cape Verde or Central African Republic or Chad or Chile or China or Colombia or Comoros or Comoro Islands or Comores or Mayotte or Congo or Zaire or Costa Rica or Cote d Ivoire or Ivory Coast or Croatia or Cuba or Cyprus or Czechoslovakia or Czech Republic or Slovakia or Slovak Republic or Djibouti or French Somaliland or Dominica or Dominican Republic or East Timor or East Timur or Timor Leste or Ecuador or Egypt or El Salvador or Eritrea or Estonia or Ethiopia or Fiji or Gabon or Gabonese Republic or Gambia or Gaza or Georgia Republic or Georgian Republic or Ghana or Gold Coast or Grenada or Guatemala or Guinea or Guam or Guiana or Guyana or Haiti or Honduras or India or Maldives or Indonesia or Iran or Iraq or Jamaica or Jordan or Kazakhstan or Kazakh or Kenya or Kiribati or Korea or Kosovo or Kyrgyzstan or Kirghizia or Kyrgyz or Kirghiz or Kirgizstan or Lao PDR or Laos or Latvia or Lebanon or Lesotho or Basutoland or Liberia or Libya or Lithuania or Macedonia or Madagascar or Malagasy or Malaysia or Malaya or Malay or Sabah or Sarawak or Malawi or Nyasaland or Mali or Marshall Islands or Mauritania or Mauritius or Agalega Islands or Mexico or Micronesia or Middle East or Moldova or Moldovia or Moldovian or Mongolia or Montenegro or Morocco or Ifni or Mozambique or Myanmar or Myanma or Burma or Namibia or Nepal or Netherlands Antilles or New Caledonia or Nicaragua or Niger or Nigeria or Mariana Islands or Oman or Muscat or Pakistan or Palau or Palestine or Panama or Paraguay or Peru or Philippines or Philipines or Phillipines or Phillippines or Romania or Rumania or Roumania or Russia or Russian or Rwanda or Ruanda or Saint Kitts or St Kitts or Nevis or Saint Lucia or St Lucia or Saint Vincent or St Vincent or Grenadines or Samoa or Samoan Islands or Navigator Island or Navigator Islands or Sao Tome or Senegal or Serbia or Montenegro or Seychelles or Sierra Leone or Slovenia or Sri Lanka or Ceylon or Solomon Islands or Somalia or Somaliland or Sudan or Suriname or Surinam or Swaziland or Syria or Tajikistan or Tadzhikistan or Tadjikistan or Tadzhik or Tanzania or Thailand or Togo or Togolese or Tonga or Trinidad or Tobago or Tunisia or Turkey or Turkmenistan or Turkmen or Uganda or Ukraine or Uruguay or USSR or Soviet Union or Union of Soviet Socialist Republics or Uzbekistan or Uzbek or Vanuatu or New Hebrides or Venezuela or Vietnam or VietNam or West Bank or Yemen or Yugoslavia or Zambia or Zimbabwe or Rhodesia).
