# Supplementary material for: Experiences, needs and priorities of family caregivers of people with severe mental health conditions in low- and middle-income countries: A systematic review of qualitative studies
Source: Glob Ment Health (Camb). 2026 May 13;13:e115. doi: 10.1017/gmh.2026.10224 (PMC13244235; doi:10.1017/gmh.2026.10224)
Supplement: Peer review history

# Reviewer report: Review: Experiences, needs and priorities of family caregivers of people with severe mental health conditions in low- and middle-income countries: A systematic review of qualitative studies — R0/PR2

**DOI:** 10.1017/gmh.2026.10224.pr2

This manuscript synthesizes qualitative evidence on the experiences, needs, and priorities of caregivers of people with severe mental health conditions in low- and middle-income countries (LMICs). The topic is important and the thematic findings are informative and compelling, with clear potential to inform service planning and family support in settings where formal care is limited. However, I recommend major revisions because several core elements need tightening for conceptual coherence and methodological transparency. Most importantly, the manuscript’s scope and terminology appear misaligned (e.g., “severe mental health conditions” vs the diagnostic groups actually included; “family caregivers” vs inclusion of broader informal caregivers), and there are internal inconsistencies between eligibility criteria and the study characteristics reported later. In addition, the methods require clearer reporting (e.g., screening process, analytic approach), and the results section should use consistent terminology and could benefit from a compact table linking themes to exemplar quotations. Finally, tables/figures and reference formatting do not currently meet journal requirements and require substantial reformatting. Addressing these issues would substantially improve the clarity, credibility, and usability of the review’s conclusions.

102 Sentence needs a reference.

103 Before talking about the mental health treatment gap for MHCs, it is important to describe THE existing lines of treatment (particularly HICs vs LMICs).

108-110 Good, but evidence from HICs where family care is not the primary resource can not illustrate the amount of burden on families in LMICs. That should be clearly highlighted.

The second paragraph would benefit from the restructuring. At the moment, there is a lot of back-and-forth, starting with limited resources in LMICs, to the family burden (in HICs), to family interventions in HICs, back to LMICs. No need to introduce HIC’s abbreviation twice.

120-122: First, state the existing reviews and the evidence, then highlight their limitations.

122-123 Now you state that no systematic review has synthesized qualitative evidence on caregiver experiences and support needs across a broad range of LMICs. What do you mean by a broad range of LMIcs? Do previous systematic reviews exist at all, and if so, how exactly is their scope limited?

This connection between the existing evidence, 1) what we know from existing reviews; 2) what their limitations are; 3) what we don’t know from previous reviews, and 4) how this review will fill in that gap, needs to be addressed in more detail and precision.

The introduction is not written in depth (with regard to the connection between the existing evidence, its limitations, and the current paper), and the relevant concepts are not introduced with sufficient detail or structure. Research question and objectives/aims are not clearly stated. Major revisions of format and context are needed.

132 prospectively registered

135 families* caregiving

136-137 Severe MHCs included affective and non-affective psychosis. This is problematic as then the scope and title of this work should be limited to ‘’affective and non-affective psychoses’' and not severe MHCs in general (including PTSD, severe MDD, BPD...).

138 ‘’...other informal (unpaid) caregiver involved in providing care.‘’ This is problematic, as the scope is broader than the title and the rest of the paper (e.g., a neighbor, community member, or local shaman could be an informal caregiver, which is NOT a family member).

142The 7th February is already a 1-year-old search. Running an updated search is advised.

148 Here you explicitly differentiate between family AND caregivers, but in the title and throughout the text, you write family caregivers only.

154 ‘’analysis methods’' is vague.

161 But was the screening double-rated (which it SHOULD) or single-rated by 5 different reviewers?

208-210 Here you state that you included studies with participants

diagnosed with schizophrenia... (13.9%) included individuals with bipolar disorder or those broadly categorized under severe mental illness (13.9%). This is not in line with what you state in lines 136-137

Sometimes you report percentages as numbers and sometimes in words; keep it consistent (e.g., report the percentage of X as a number, and in parentheses, N = Y).

220 Don’t introduce this abbreviation since you won’t use it again. Interpretative Phenomenological Analysis (IPA).

223 Report percentages and N of the majority of studies...

3.2. Overview of themes - the general problem is that you use the words families and caregivers " intertwined, which can ot be mixed up if you include non-family caregivers in your review. This problem stems from earlier, but in the results section, it becomes particularly relevant.

322-325 These are the strong points, and an example quotation is important here.

356-370 The paragraph on stigma would benefit from additional example quotes.

384-385 Reference is in italic font by accident.

In general, the overview of the themes is informative, well-written, meaningful, and touching! If space allows, an overview table with the points and main quotes for each theme would be beneficial.

Discussion: Strengths and Limitations are underdeveloped.

References are not consistently formatted according to the required journal style (Cambridge A).

Tables and Figures are badly organized. They do not follow the journal requirements (no colour, single Table/figure per page, etc.). Line/page spacing is sometimes off (e.g., PRISMA Flow diagram). Not clear when one table ends, and another begins. That being said, table titles are not placed properly. Pages 28-41 are not readable. Major revisions are required here.

Text as a whole needs to be proofread again and formatting mistakes (e.g., missing spaces before parentheses, double spaces,

# Reviewer report: Review: Experiences, needs and priorities of family caregivers of people with severe mental health conditions in low- and middle-income countries: A systematic review of qualitative studies — R0/PR3

**DOI:** 10.1017/gmh.2026.10224.pr3

**Authors:** Racheal Alinaitwe

**Affiliations:** Psychiatry, Makerere University College of Health Sciences, Uganda

An excellent review of a critical area in mental health. Discusses the review regarding the family as a unit that attempts to bridge the treatment gap in global mental health

Abstract: well-detailed, coherent, and well-written; captivates the reader.

Impact summary: Excellently done

Introduction: lines 108-110; it would have been good to highlight some of these caregiver burdens. Little is said about the experiences, needs and priorities of these caregivers in the introduction instead, more focus is put on the interventions.

Methods: clearly outlined and standard reporting guidelines (ENTREQ) were followed

Data extraction: lines 161to 165; titles and abstracts were reviewed by 5 reviewers, but disagreements were resolved through consultation with a third reviewer. The authors should clarify the number of reviewers, possibly there’s a statement missing hence the confusion

Strategy for data synthesis: lines 180 to 185, it would be important to indicate which sections of the included articles were uploaded/exported into NVIVO and what was coded; was it all sections labelled findings/results, was it findings and discussion? What exactly was coded?

Author reflexivity is rather vague. The authors need to elaborate on how the diverse professional backgrounds shaped the generation of the findings.

Familial commitment to care; you have reported the female caregiver burden under this, though it might need to be presented as a separate theme to give it the weight it deserves

Discussion: places the findings of the review in context with previous literature

# Editor report: Recommendation: Experiences, needs and priorities of family caregivers of people with severe mental health conditions in low- and middle-income countries: A systematic review of qualitative studies — R0/PR4

**DOI:** 10.1017/gmh.2026.10224.pr4

**Affiliations:** Centre for Rural Health, University of KwaZulu-Natal, South Africa

Dear Dr. ENKOYEE

Following inputs from two independent reviewers, a decision has been reached for your submission titled “Experiences, needs, and priorities of family caregivers of people with severe mental health conditions in low and middle-income countries: a systematic review of qualitative studies”. The recommended outcome from the review was “Major Revision”. Please read reviewer comments carefully, and address each point, while documenting your response to each point raised before re-submitting your manuscript. We are looking forward to receiving your revised submission.

Best regards,

# Editor report: Decision: Experiences, needs and priorities of family caregivers of people with severe mental health conditions in low- and middle-income countries: A systematic review of qualitative studies — R0/PR5

**DOI:** 10.1017/gmh.2026.10224.pr5

**Affiliations:** London School of Hygiene & Tropical Medicine, United Kingdom of Great Britain and Northern Ireland

No accompanying comment.

# Reviewer report: Review: Experiences, needs and priorities of family caregivers of people with severe mental health conditions in low- and middle-income countries: A systematic review of qualitative studies — R1/PR7

**DOI:** 10.1017/gmh.2026.10224.pr7

Thank you for your thorough and thoughtful revision of the manuscript and for the detailed, point-by-point responses to the review comments. I appreciate your taking the time to address the conceptual, methodological, and reporting issues raised in the first review.

The revised manuscript shows substantial improvement. In particular:

* The introduction is now much clearer and better structured, with a more precise articulation of existing evidence, its limitations, and the specific contribution of the current review. The research aim and gap are now well-defined.

* The issues related to scope and terminology (e.g., definition of severe mental health conditions and clarification of “family caregivers”) have been adequately resolved, improving conceptual coherence.

* The methods section has been strengthened, with clearer reporting of eligibility criteria, analytic approaches, and screening procedures.

* The results section now uses more consistent terminology, and the addition of a supplementary table with themes and illustrative quotations is an appropriate and effective solution given word limits.

* The discussion, including strengths and limitations, has been expanded and is now more balanced.

* Formatting, referencing, and structural issues appear to have been carefully addressed.

While some methodological choices (e.g., single screening, no search updates) remain limitations, they are now transparently acknowledged and reasonably justified in the manuscript.

Overall, I am satisfied that my major concerns have been addressed, and I believe the manuscript now meets the standards for publication. I therefore recommend acceptance in its current form.

# Reviewer report: Review: Experiences, needs and priorities of family caregivers of people with severe mental health conditions in low- and middle-income countries: A systematic review of qualitative studies — R1/PR8

**DOI:** 10.1017/gmh.2026.10224.pr8

**Authors:** Racheal Alinaitwe

**Affiliations:** Psychiatry, Makerere University College of Health Sciences, Uganda

The authors have adequately addressed all the comments I previously raised. I must commend them on the great work. The manuscript reads much better.

# Editor report: Recommendation: Experiences, needs and priorities of family caregivers of people with severe mental health conditions in low- and middle-income countries: A systematic review of qualitative studies — R1/PR9

**DOI:** 10.1017/gmh.2026.10224.pr9

**Affiliations:** Centre for Rural Health, University of KwaZulu-Natal, South Africa

Dear Dr. ENKOYEE,

Following your revisions in response to peer review critiques and inputs, we have the pleasure to inform you that your paper has been accepted for publication. The editorial office will be in touch with next steps shortly.

Sincerely,

Prof. André Janse van Rensburg

Cambridge Prisms: Global Mental Health

Manuscript ID: GMH-2025-0458.R1

Manuscript Type: Review

Manuscript Title: Experiences, needs, and priorities of family caregivers of people with severe mental health conditions in low and middle-income countries: a systematic review of qualitative studies

# Editor report: Decision: Experiences, needs and priorities of family caregivers of people with severe mental health conditions in low- and middle-income countries: A systematic review of qualitative studies — R1/PR10

**DOI:** 10.1017/gmh.2026.10224.pr10

**Affiliations:** London School of Hygiene & Tropical Medicine, United Kingdom of Great Britain and Northern Ireland

No accompanying comment.
